# The TNKS/PI31–proteasome axis as a central node in TDP-43 neuropathies

**DOI:** 10.1186/s12929-026-01296-1

**Published:** 2026-11-04

**Authors:** Pitchapa Bubphachat, Pritha Majumder, I.-Fang Wang, Jeffrey K. Huang, Tzu-Jen Kao, Yi-Chao Lee, Che-Kun James Shen, Guan-Chou Chen, Po-Chih Chen, Chi-Chen Sarah Huang

**Affiliations:** 1 Ph.D. Program in Medical Neuroscience, College of Medical Science and Technology, Taipei Medical University and National Health Research Institutes, Shuang-Ho Campus, New Taipei City, 235 Taiwan; 2 International Master Program in Medical Neuroscience, College of Medical Science and Technology, Taipei Medical University, Shuang-Ho Campus, New Taipei City, 235 Taiwan; 3 School of Medicine, College of Medicine, Taipei Medical University, Taipei, 110 Taiwan; 4 TMU Research Center of Neuroscience, Taipei Medical University, Taipei, 110 Taiwan; 5 Department of Biology, Georgetown University, Washington, DC 20057 United States; 6 Institute of Molecular Biology, Academia Sinica, Taipei, 115 Taiwan; 7 Department of Neurology, Shuang-Ho Hospital, Taipei Medical University, New Taipei City, 235 Taiwan; 8 Taipei Neuroscience Institute, Taipei Medical University, New Taipei City, 235 Taiwan; 9 Department of Neurology, School of Medicine, College of Medicine, Taipei Medical University, Taipei, 110 Taiwan

**Keywords:** TDP-43, ALS, TNKS, PI31, Proteasome activity, Axon transport, ADP-ribosylation, Live cell imaging, Protein homeostasis

## Abstract

**Background:**

Pathological aggregation of Tar DNA-binding protein 43 (TDP-43) and disruption of neuronal protein homeostasis are defining features of amyotrophic lateral sclerosis (ALS), frontotemporal lobar degeneration (FTLD), and related TDP-43 proteinopathies. Efficient axonal transport of proteasomes is essential for maintaining local protein quality control in neurons. Proteasome inhibitor 31 (PI31) regulates both 26S proteasome assembly and dynein-mediated transport of 20S proteasomes. Although Tankyrase 1/2 (TNKS)-mediated ADP-ribosylation of PI31 promotes 26S proteasome assembly, its role in proteasome transport and its contribution to TDP-43 pathology remain unclear.

**Methods:**

Live-cell imaging was performed to examine 20S proteasome transport in primary cortical and motor neurons from wild-type and Prp-TDP-43^A315T^ transgenic mice. Protein interactions among TDP-43, TNKS, and PI31 were analyzed by immunoprecipitation and Förster resonance energy transfer (FRET). Insoluble protein accumulation and colocalization were assessed by RIPA/urea fractionation, Western blotting, and immunofluorescence. Proteasome assembly and activity were evaluated using native gel electrophoresis and a 20S proteasome-specific fluorogenic substrate. TDP-43 aggregation was examined by semi-denaturing detergent agarose gel electrophoresis (SDD-AGE).

**Results:**

In cell models expressing TDP-43-FL, TDP-43-A315T, or TDP-25 and in Prp-TDP-43^A315T^ transgenic mice, pathological TDP-43 species formed distinct aggregates, ranging from oligomers to prominent cytoplasmic aggregates, yet all reduced proteasome activity, impaired axonal proteasome transport, and caused the accumulation of polyubiquitinated proteins, PI31, 20S proteasomes, and TDP-43 in the proximal axon and cell body. Pathological TDP-43 sequestered TNKS and proteasome components into insoluble fractions, thereby limiting the availability of free and soluble TNKS for interaction with PI31 and subsequent PI31 ADP-ribosylation, ultimately impairing 19S/20S proteasome assembly. Notably, disruption of the TNKS–PI31 interaction or inhibition of TNKS activity recapitulated the proteasome dysfunction induced by pathological TDP-43, supporting a role for the aberrant TNKS–TDP-43 interaction and consequent impairment of TNKS-mediated PI31 ADP-ribosylation in TNKS/PI31/proteasome axis dysfunction.

**Conclusions:**

Pathological TDP-43 disrupts proteasome function by impairing the TNKS/PI31/proteasome axis, suggesting that this axis may represent a potential therapeutic target for TDP-43 proteinopathies. Selectively restoring this pathway may enhance pathological TDP-43 clearance while minimizing nonspecific proteasome activation, thereby providing a more precise and safer therapeutic strategy for ALS and FTLD.

**Supplementary Information:**

The online version contains supplementary material available at https://doi.org/10.1186/s12929-026-01296-1.

## Background

Numerous neurodegenerative diseases are characterized by the accumulation of abnormally misfolded and ubiquitinated proteins in the form of insoluble aggregates, accompanied by a loss of protein homeostasis, ultimately leading to progressive neuronal death and reactive gliosis [1, 2]. Decreased proteasome function has been reported to be the major cause of abnormal accumulations of these misfolded proteins. But whether impaired proteasome function is the cause or the consequence of these accumulated aggregation protein remain an elusive issue [3]. Ubiquitinated Inclusions (UBIs) is the pathological hallmark of Amyotrophic lateral sclerosis (ALS) and Frontotemporal lobar degeneration (FTLD), in which Tar DNA binding protein 43 (TDP-43)-positive ubiquitinated inclusions is discovered as the major pathological components of these UBIs in 95% of ALS patients and 45% Frontotemporal lobar degeneration with ubiquitin-positive inclusion (FTLD-U) patients [4–6]. TDP-43 protein is a 414 amino acid protein encoded by the TARDBP gene which is highly conserved in various species and ubiquitously expressed in all tissues including brain [7–9]. The N-terminus RNA‐recognition motifs (RRM I and RRM II) of TDP‐43 are important for DNA/ RNA-binding [7], while the glycine-rich C‐terminal region of TDP‐43 is required for self‐regulation, exon skipping and pre-mRNA splicing inhibitory reactions [9, 10]. TDP-43 contains both nuclear localization and nuclear export signals that facilitate its nucleocytoplasmic shuttling. Under physiological conditions, TDP-43 is predominantly localized in the nucleus, where it regulates RNA processing and metabolism. A smaller cytoplasmic pool of TDP-43 contributes to the regulation of mRNA stability, transport, and translation. Dysregulation of TDP-43 nucleocytoplasmic trafficking can lead to cytoplasmic accumulation and aggregation, accompanied by loss of its normal nuclear functions in disease states [11–13]. The pathological TDP-43 protein is hyperphosphorylated, polyubiquitylated, cleaved into C-terminal 35 kDa (TDP-35) and 25 kDa (TDP-25) fragments, long protein half-lives, and sequestered into cytosol to form detergent-insoluble cytosolic aggregates in the central nervous systems, including hippocampus, cortex, and spinal cord motor neuron [5]. In FTLD-U and ALS patients, accumulation of TDP-43 within ubiquitin-positive insoluble aggregates implies that the impaired ubiquitin proteasome metabolism cascades are involved in TDP-43-associated pathology [5]. It has been demonstrated that motor neuron specific disruption of proteasome, rather than autophagy, fundamentally governs the progression of ALS-linked pathologies, including ALS hallmarks TDP-43 aggregates formation and locomotor dysfunction accompanied by progressive motor neuron loss and gliosis, thus implicating that enhancement of proteasome activity would be a promising therapeutic strategies for ALS patients [14, 15]. In cellular and murine models of ALS, enhancement of proteasome activity by small molecules, pyrazolone, shows prominent therapeutic effect, strongly replicating a potential role of proteasome for the therapeutic target of ALS [16]. However, proteasome activation without selectively targeting specific substrates may lead to undesirable side effects and complex cellular consequences. Therefore, developing approaches to restore proteasome regulatory mechanisms selectively disrupted by pathological TDP-43, rather than broadly activating the entire proteasome, are likely to minimize off-target effects and offer a more effective approach to ameliorate the pathological hallmarks of TDP-43 proteinopathies.

It has been well demonstrated that the 26S proteasome consists of the 19S regulatory ATPase/non-ATPase subunits and the 20S catalytic core which is made up with α7β7β7α7 subunits to serves for selectively and specifically substrate degradation. Of the seven β subunits, only β1, β2, and β5 possess proteolytic activity and exhibits caspase-like, trypsin-like, and chymotrypsin-like activities, respectively. Proteasome activity is regulated at multiple levels, including the expression of proteasome subunits, assembly of the 19S and 20S complexes, post-translational modifications, and the utilization of different regulatory proteins [17–21]. Among these regulatory mechanisms, the ADP-ribosylation of the proteasome inhibitor 31 (PI31) mediated by poly ADP-ribose polymerase (PARP), namely tankyrase 1 (TNKS1 or PARP5a) or tankyrase 2 (TNKS2 or PARP5b), plays a central role in coordinating the assembly of the 19S and 20S proteasome and thereby providing critically regulation of 26S proteasome activity [22]. Assembly of the 26S proteasome is mediated in part by the C-terminal HbYX motifs present in several 19S ATPases, including Rpt2, Rpt3, and Rpt5, which dock into pockets in the 20S α-ring to promote gate opening and substrate entry [17, 23, 24]. Based on this mechanism, previous studies proposed that PI31 interact with the 20S proteasome through a similar HbYX motif located in its C-terminal region, and that mutation of this motif could affect proteasome activity [25–27]. Recent cryo-electron microscopy studies have shown that PI31 binds the interior of the 20S cylinder via its proline-rich C-terminal region and inhibits proteasome activity by competing with 19S ATPases for 20S binding and inserting into the 20S central cavity to directly engage catalytic sites [28–30]. Under this inhibitory conditions, the chaperons p27 and S5b tether the 19S regulatory particle, further preventing the assembly of the 19S and 20S subunits [22]. Once TNKS directly interacts with PI31 at its canonical TNKS—binding motif (RxxGxGxE/D) and catalyzes PI31 ADP-ribosylation, it transfers an ADP-ribose moiety from NAD+ to PI31 and releases PI31 from the 20S core, thus relieving the inhibitory effect of PI31 on 26S proteasome activation [22]. In this configuration, PI31 is sequestered by p27/S5b, thereby releasing the 19S regulatory particle from p27/S5b. Subsequently, the 19S regulatory particle assembles with the 20S to form the active 26S proteasome [22]. Taken together, these results demonstrate that the TNKS-PI31 axis constitutes a conserved proteasome-regulatory cascade that plays a critical role for modulating 26S proteasome activity. In addition to regulating 26S proteasome assembly and activation, PI31 also functions as an adaptor for axonal transport of 20S proteasome [31]. PI31 facilitates the interaction between proteasomes and cellular motors by directly binding to dynein light chain LC8-type proteins (DYNLL1/2), which promotes the fast axonal transport of proteasomes between the soma and neuron terminals [31]. The targeted delivery of proteasomes to sites of protein degradation is essential for maintaining proteostasis and preserving synaptic integrity in neurons [31, 32]. While TNKS-mediated PI31 ADP-ribosylation has been identified as a key mechanism regulating 26S proteasome activity, it remains unknown whether TNKS also regulates PI31-dependent proteasome transport within axons. Given the critical importance of proteasome spatial distribution for neuronal protein homeostasis, elucidating this potential TNKS/PI31/proteasome regulatory axis is an important strategy that may provide a potential therapeutic target for the future development of specific proteasome-activating drugs.

Our study is the first to reveal that TNKS is not only crucial for PI31-dependent 20S proteasome activation, but also essential for proteasome axonal transport under normal condition. When TNKS activity is inhibited, PI31-mediated proteasome motility is significantly impaired, leading to the accumulation of insoluble 20S proteasome, TDP-43, and PI31 in the cell body and proximal axons, reflecting the characteristic pathological features of ALS. While in the Prp-TDP-43^A315T^ transgenic (Tg) mice used in our study overexpressing the familial ALS-associated TDP-43 mutation and recapitulating ALS-like pathologies, the coordinated TNKS–PI31–proteasome signaling cascade is disrupted by pathological TDP-43 through sequestration of TNKS into insoluble fractions, thus impairing axonal transport of 20S proteasomes and ultimately reducing 26S proteasome activation and impairing local protein homeostasis. Overall, we propose a novel model to explain how pathological TDP-43 specifically impairs TNKS/PI31/proteasome function and disrupts protein homeostasis in TDP-43-related neuropathies. Our findings shed new light on exploring a specific signaling cascades to restore the TNKS–PI31–proteasome regulatory mechanism. Blocking the aberrant and excessive binding between TNKS and TDP-43 may offer a potential strategy for restoring proteaosome function and ameliorating the pathological hallmarks of TDP-43 proteinopathies. This targeted approach minimizes potential off-target effects and adverse side effects from non-specific proteasome activation, while simultaneously improving the pathological features of TDP-43 protein disorders.

## Materials and methods

B6.Cg-Tg (Prnp-TARDBP*A315T)95Balo/J mice—Transgenic mice expressing mutant human TDP-43-A315T in the brain and spinal cord under the control of the PrP (Prion) promoter (B6.Cg-Tg (PrnpTARDBP*A315T)95Balo/J mice) were obtained from the Jackson Laboratory Repository, JAX stock no. 010700) [33, 34]. We backcrossed Prp- TDP-43^A315T^ Tg mice with C57BL/6 mice to obtain heterozygous Prp-TDP-43^A315T^ Tg mice and their non-transgenic wild-type littermates (Non-Tg wild-type control mice) on the same genetic background. All animals were kept in an air-conditioned facility under a 12 h light and 12 h dark cycle, with free access to food and water. To prevent gastrointestinal complications,, from day 30 after birth until the end of the experiment, the mice were fed a high-fat gel diet (DietGel Boost, ClearH2O, Westbrook, ME, USA), a purified high-calorie dietary supplement, placed in a cup hanging on the cage. This ensured that animals showing motor impairment at disease onset could easily access nutrition and hydration. The mice were weighed and observed daily by trained animal technicians for the appearance of ALS-related symptoms until they met the humane endpoint, defined as severe motor dysfunction. The median lifespan of male Prp-TDP43^A315T^ mice was 96 ± 18 day, and the median lifespan of female mice was 196 ± 15 days [33, 35]. Tissues from all animals were collected after the onset of ALS-like symptoms, defined as severe motor dysfunction accompanied by a 15–20% loss of body weight. Animal handling and experimental procedures followed the 3Rs principles, and animals used in this study were treated according to the guidelines of the Taipei Medical University Institutional Animal Care and Use Committee (IACUC) under permit number LAC2023–0194, SHLAC2023-0073 and SHLAC2024-0126.

*Antibody*—All antibodies used in this study are listed in Table 1.

**Table 1:** List of antibodies used in this study

Ab name	Brand #Cat. No	Titer	Species	M.W (kDa)
GFP	Thermo Fisher Scientific #A11122	1:3000	Rabbit	24–26
TDP-43	Proteintech #10782-2-AP	1:2000	Rabbit	43
TDP-43	Proteintech #67345-1-Ig	1:2000	Mouse	43
Phospho-TDP43 (Ser409/410)	Proteintech #23309-1-AP	1:2000	Rabbit	43
α-tubulin	Proteintech #11224-1-AP	1:10,000	Rabbit	50–55
β-actin	Genetex #GTX109639	1:1000	Rabbit	42
GAPDH	Proteintech # 60004-1-Ig	1:20,000	Mouse	36
ChAT	Proteintech #20747-1-AP	1:1000	Mouse	47–50/66–70
Flag	Proteintech #66008-3-Ig	1:2000	Mouse	
PI31	Santa Cruz #sc-136416	1:1000	Rabbit	31
TNKS	Proteintech #18030-1-AP	1:2000	Rabbit	150
ADP-Ribose	Cell Signaling Technology #83732S	1:1000	Rabbit	
PSMD4	Proteintech #12441-1-AP	1:1000	Rabbit	50
PSMC6	Proteintech #15839-1-AP	1:5000	Rabbit	42
PMSA1	Proteintech #3632-1	1:5000	Rabbit	30
PSMC5 (Rpt6)	Enzo #BML-PW9265-0100	1:1000	Mouse	48
Pan 20S proteasome (α5/α7, ß1, ß5, ß5i, ß7)	Abcam # 22673	1:1000	Rabbit	25–35

*Cell culture and transfection—Neuro 2a (N2a)* cells (Bioresource Collection and Research Center, Hsinchu, Taiwan) were cultured in Eagle’s minimum essential medium (Thermo Fisher Scientific, Carlsbad, CA, USA), and *NSC34* (Bioresource Collection and Research Center) were cultured in Dulbecco’s modified Eagle’s medium (Thermo Fisher Scientific), supplemented with 10% (v/v) fetal calf serum and 1% penicillin–streptomycin (Thermo Fisher Scientific). *For primary cortex culture*, embryonic cerebral cortices derived from E16.5 mouse brain were isolated and enzymatically dissociated using papain. The tissue was then gently triturated by pipetting in neuronal culture medium consisting of Neurobasal™ Plus Medium (Thermo Fisher Scientific) supplemented with 1× B-27™ (Gibco) to generate a single-cell suspension. Cortical neurons were seeded on glass coverslips coated with poly-l-lysine (50 μg/mL; Sigma-Aldrich, Burlington, MA, USA) and maintained at 37 °C in a humidified incubator with 5% CO₂. *For primary motor culture*, embryonic spinal cord derived from E12.5 mouse were isolated and enzymatically dissociated using papain. The tissue was then gently triturated by pipetting in neuronal culture medium consisting of Neurobasal™ Medium (Thermo Fisher Scientific) supplemented with 1× B-27™ (Thermo Fisher Scientific), 0.05 mM L-Glutamic acid (Sigma-Aldrich), and 2 mM L-Glutamine (Gibco) to generate a single-cell suspension. Motor neurons were seeded on glass coverslips coated with poly-l-lysine (50 μg/mL; Sigma-Aldrich) and laminin (20ug/ml; Thermo Fisher Scientific), and then maintained at 37 °C in a humidified incubator with 5% CO₂. All experiments were performed by transfecting equal amounts of plasmid DNA into the same number of cells. Transfection was performed using Lipofectamine 2000 (Thermo Fisher Scientific) at a 1:1 DNA:Lipofectamine ratio in Opti-MEM for 4 h, followed by replacement with fresh culture medium, and cells were harvested 48 h after transfection for subsequent analyses..

*Plasmid constructs*—TDP-43 related mutants were generated using GFP–TDP-43 plasmid [14] as template. Missense mutations were introduced with the QuikChange Site-Directed Mutagenesis Kit (Stratagene, La Jolla, CA, USA). The cDNA derived from PCR with specific primers was excised by restriction enzymes, and then ligated into pEGFP-C1, pEYFP-N1, pECFP-C1, pcDNA™3.1/myc-His A (Thermo Fisher Scientific) or pCMV-Flag-tag2 (Addgene, Cambridge, MA, USA) vectors by T4 DNA ligase (Takara Bio, San Jose, CA, USA). The detail information for plasmid construction were listed in Table 2.

**Table 2:** Primer sequences used for PCR or constructions of various plasmids

Plasmid name	Template	Vector	Forward-primer/reverse-primer	Restriction enzyme
GFP-TDP-25 (amino acids 175–414)	GFP–TDP-43	pEGFP-C1	5’-CGGAAGCTTCGAATTCTAAGCAAAGCCAA-3’/ 5’-CAGGTACCCTACATTCCCCAGCCAGA-3’	HindIII/KpnI
GFP–TDP-43-A315T	GFP–TDP-43	pEGFP-C1		
GFP–TDP-43-FL	GFP–TDP-43	pEGFP-C1	The cDNA fragments were excised from templates by restriction enzymes and then ligated into the vector by T4 DNA ligase	Xho1 and Xba1
GFP–TDP-43-A315T	GFP–TDP-43-A315T	pEGFP-C1		Xho1 and Xba1
GFP-TDP-25 (amino acids 175–414)	GFP-TDP-25	pEGFP-C1		Xho1 and Xba1
GFP-TDP-43-TBD (H166A and I168A)	GFP-TDP-43-TBD	pEGFP-C1	*TDP-43-TBD-AA-F primer* AAA GTA ATG TCA CAG CGA GCT ATG GCT GAT GGA CGA TGG TGT GAC *TDP-43-TBD-AA-R primer* GTC ACA CCA TCG TCC ATC AGC CAT AGC TCG CTG TGA CAT TAC TTT	Site-Directed Mutagenesis
YFP-TNKS	hTNKS	pEYFP-N1	5’-CCC A’AG CTT GGG ATG GCG GCG TCG CGT-3’/ 5’-GCA GAG CAG AAG ACC CCA’ CCG GTC-3’	HindIII/AgeI
Flag-TNKS	hTNKS	pCMV-flag-tag2	5’-CCA’ AGC TTG GCG GCG TCG CGT CGC-3’/ 5’-GCA GAG CAG AAG ACC TAG CCC’ TCG AGG G-3’	HindIII-HF/XhoI
CFP-PI31	hPI31	pECFP-C1	5’-CCC’T CGA GGG GCG GGC CTG GAG GTA-3’/ 5’-TAC GAT GAC ATG TAC CTG TGA TGC T’CT AGA GC-3’	XhoI/XbaI
MYC-PI31	hPI31	pcDNA™3.1/myc-His A	5’-GGGGTAC’CCC ATG GCG GGC CTG GAG-3’/ 5’-GAT GAC ATG TAC CTG CCG C’TC GAG CGG-3’	KpnI/XhoI

*Immunoprecipitation*—Protein lysates from TDP-43-A315T mutant-transfected mouse neuroblastoma N2a cells, mouse motor neuron-like hybrid NSC34 cells (Cold Spring Biotech. Corp., Xizhi, New Taipei City, Taiwan), brain or spinal cord tissue lysates from Non-Tg wild-type control littermate or Prp-TDP-43^A315T^ Tg mice are lyzed in lysis buffer (50 mM Tris–HCl (pH 7.8), 150 mM NaCl, 0.1% Nonidet P-40 and cOmplete™ Protease Inhibitor Cocktail (Roche, Cat# 04693159001)). Whole-cell lysates were incubated with the indicated antibodies at 4 °C overnight. The resulting immunocomplexes were then captured with Protein A–Sepharose for 1 h at 4 °C. After incubation, the beads were gently washed three times with the same buffer, and the bound proteins were analyzed by Western blot with indicated antibodies.

*Sequential biochemical fractionation and immunoblotting*—We performed sequential extractions to examine the solubility profile of TDP-43. For all cell culture experiments, equal numbers of cells were transfected with identical amounts of plasmid DNA. Collected cell lysate were washed with PBS twice and lyzed in RIPA buffer (50 mM Tris–HCl (pH 8.0), 150 mM NaCl, 1% NP-40, 5 mM EDTA, 0.5% sodium deoxycholate, 0.1% SDS) supplemented with 1 mM PMSF and protease/phosphatase inhibitors. Lysates were centrifuged at 100,000 × g for 30 min at 4 °C, and the supernatants were collected as the RIPA-soluble fractions. Pellets were rewashed twice in RIPA buffer and recentrifuged, after which the supernatant was discarded. The final pellets were solubilized by sonication in urea buffer (7 M urea, 2 M thiourea, 4% CHAPS, 30 mM Tris–HCl (pH 8.5) and centrifuged at 100,000 × g for 30 min at 22 °C. The resulting supernatants were designated as RIPA-insoluble/urea-soluble fractions.

Brain and spinal cord tissues from Prp-TDP-43^A315T^ transgenic mice were collected after disease onset. Post-mortem brain and spinal cord tissues were dissected, weighed, and sequentially extracted. Tissues were homoginized in a ratio of 5 mL/g (volume/weight) with RIPA buffer (5× v/w; 50 mM Tris–HCl (pH 8.0), 150 mM NaCl, 1% NP-40, 5 mM EDTA, 0.5% sodium deoxycholate, 0.1% SDS) supplemented with 1 mM PMSF and protease/phosphatase inhibitors. Lysates were centrifuged at 100,000 × g for 30 min at 4 °C, and the supernatants were collected as the RIPA-soluble fractions. Pellets were rewashed by sonication in myelin flotation buffer (10 mM Tris–HCl (pH 7.5), 5 mM EDTA, 1% (v/v) Triton-X-100 and 0.5 M NaCl, and 30% sucrose), and re-centrifuged, after which the supernatant was discarded. Then the pellets were rewashed twice by sonication in RIPA buffer and recentrifuged, after which the supernatant was discarded. The final pellets were solubilized by sonication in urea buffer (2× v/w; 7 M urea, 2 M thiourea, 4% CHAPS, 30 mM Tris–HCl (pH 8.5) and centrifuged at 100,000 × g for 30 min at 22 °C [29–31]. The resulting supernatants were designated as RIPA-insoluble/urea-soluble fractions. Protein concentrations of RIPA-soluble fractions were determined using a BCA assay. As described previously, equivalent amounts of total protein (~ 20 μg) from the RIPA-soluble fraction were loaded per sample, while the loading amount of the urea fraction was 1.5 times the volume of the corresponding RIPA soluble protein sample [36–38]. Extracted proteins were separated on 10% or 4–20% gradient Tris–glycine SDS-PAGE, transferred to PVDF membranes, and probed with the indicated primary and secondary antibodies [5]. Consistent to previous studies, the internal control proteins such as GAPDH, α-tubulin, or β-actin could only be detected in the RIPA-soluble fraction, as their expression levels remain stable and these proteins are not extracted into the urea-soluble fraction [36–38].

For total protein extraction, whole-cell debris or pellets were solubilized by sonication in urea buffer (2 × volume/weight; 7 M urea, 2 M thiourea, 4% CHAPS, 30 mM Tris–HCl, pH 8.5) and centrifuged at 100,000 × g for 30 min at 22 °C [29–31]. The resulting supernatants were designated as the RIPA-insoluble/urea-soluble fractions. Protein concentrations of the RIPA-soluble fractions were determined using a BCA assay, and equal amounts of protein were analyzed by western blot.

The protein band intensity was normalized to the corresponding loading control to correct for differences in sample loading amount. Density measurements were performed using AlphaEase software (Alpha Innotech, San Leandro, CA, USA). For the urea fractions, normalization to an internal control protein was not applied, as this fraction does not retain the control. Instead, protein intensities in the urea fraction were normalized to the corresponding protein levels in the RIPA fraction, which had been normalized to the internal control.

*Immunofluorescence staining and confocal microscopy*—N2a or NSC34 cells cultured on poly-L-lysine–coated glass coverslips and transfected with the indicated plasmids for 48 h, or primary motor neuron cultures at 7 days in vitro (DIV) derived from n Non-Tg wild-type control or Prp-TDP-43^A315T^ Tg mice and maintained on poly-L-lysine– and laminin-coated glass coverslips, were fixed with 4% paraformaldehyde prepared in ice-cold PBS at 4 °C for 20 min and then permeabilized with 0.5% Triton X-100 for 7 min at room temperature. After blocking with 10% donkey serum for 1 h at room temperature, the cells are incubated overnight at 4 °C with first antibodies. After washing, the cells are incubated at room temperature for 1.5 h with DAPI (1:500 dilution; stock concentration: 1 mg/mL; Sigma-Aldrich) together with ALEXA FLUOR fluorescent-conjugated secondary antibodies (Thermo Fisher Scientific). The images were examined on a Zeiss LSM 710 confocal microscope.

*Ub*^*G76V*^*-GFP*—N2a or NSC34 cells were cultured in 6-well plates and transfected with the GFP-tagged ubiquitin mutant plasmid (Ub^G76V^-GFP, Addgene #11941) for 24 h. Ub^G76V^ -GFP fluorescence was imaged using an ImageXpress XL reader and quantified with MetaMorph software (Molecular Devices, Downingtown, PA, USA). For quantitative analysis, approximately 270 representative fields per well, covering the entire well area, Ub^G76V^-GFP fluorescence-positive cells were captured and analyzed using MetaMorph.

*MV151-labeling*—The fluorophore-coupled activity-based probe MV151 (BODIPY TMR-Ahx3L3VS) is a cell-permeable activity-based probe widely used to analyze 20S proteasome activity and enables visualization of 20S proteasome activity in living organisms [39, 40]. MV151 enters through the gate of the 20S proteasome and covalently labels the N-terminal threonine residues of the catalytic β1, β2, and β5 subunits within the lumen of the 20S core particle. Each 20S core particle can accommodate up to six MV151 molecules, thereby labeling the active catalytic sites of functional proteasomes [39]. For in-gel MV151 labeling (Fig. 2B), 20 µg of cell lysate proteins were incubated with 1 μM MV151 at 37 °C for 1 h, followed by separation on 15% SDS–PAGE. MV151-labeled active 20S proteasome β1, β2, and β5 subunits fluorescence were directly visualized on wet gel slabs using a Typhoon FLA 9000 laser scanner (GE Healthcare, Chicago, IL, USA) or iBright™ CL1500 Imaging System (Thermo Fisher Scientific) (excitation: 532 nm; emission: 560 nm).

*20S proteasome activity assay by fluorogenic peptide cleavage*—10 ug of N2a/NSC34 cells or brain/spinal cord tissue lysates were incubated for 1 h at 37 °C in 100 μL reaction mixture containing 50 mM Tris–HCl (pH 7.5), 250 mM sucrose, 5 mM MgCl₂, 0.5 mM EDTA, 2 mM ATP, 1 mM DTT, and 80 μM fluorogenic peptide substrates. Chymotrypsin-like activity of 20S proteasome was measured using Suc-Leu-Leu-Val-Tyr-AMC (Enzo Life Sciences, Long Island, NY, USA, #BML-PW9905-0001). The fluorescence intensity of AMC released after active proteasome cleavage of Suc-LLVY-AMC was measured every 5 min at 37 °C, and 20S proteasome activity was continuously monitored for a total of 60 min using a GloMax® Discover Microplate Reader (Promega, Madison, USA) (Ext =380 nm, Emi =460 nm). To eliminate baseline differences, the AMC fluorescence fold change at each time point was normalized relative to the corresponding 0-min time point, and the total amount of total cell or tissue lysate protein (10 μg) loaded in each sample was the same. To verify assay specificity, reaction mixtures were pre-incubated with MG132 (Sigma-Aldrich) prior to substrate addition as a negative control.

*Flow cytometry*—In NSC34 or N2a cells, equal numbers of cells transfected with the same amount of GFP, GFP–TDP-43-FL, GFP-TDP-43-A315T or GFP–TDP-25 plasmids were incubated with 1 μM MV151 at 37 °C for 1 h. Cells were harvested by trypsinization, pelleted, and rinsed with phosphate-buffered saline (PBS) prior to fixation in 75% ethanol for 1 h at room temperature. A total of 5,000 GFP (+) cells were sorted, and 20S proteasome activity was determined by analyzing the total fluorescence intensity of MV151, which was obtained by multiplying the total number of MV151(+) cells by their average MV151 fluorescence intensity. The 20S proteasome activity of GFP-transfected cells was set to 1, and fold changes in 20S proteasome activity in cells expressing the indicated GFP–TDP-43 constructs were determined and presented as bar graphs. MV151 fluorescence was measured on a BD FACS Calibur flow cytometer (BD Biosciences, Franklin Lakes, NJ, USA), and the resulting data were processed using WinMDI software (Joseph Trotter, La Jolla, CA, USA).

*Live cell imaging*—Primary cortical neurons, derived from E16.5 mouse brain were transfected with GFP or GFP-TDP-25 at DIV5 for 48 h and then incubated with 1 μM MV151 for another 1 h at DIV7. Imaging of primary motor neurons was carried out at DIV7. Primary motor neurons derived from E12.5 mouse spinal cord were incubated with 2 uM XAV939 (Tocris Bioscience, Minneapolis, MN, USA, #3748) for 15 h at DIV5 and then 1 μM MV151 for another 1 h at DIV6. Imaging of primary motor neurons was carried out at DIV6. Live-cell imaging was performed to examine axonal proteasome movement on a Zeiss Observer Z1 equipped with an AxioCam MRm camera and temperature/CO₂ control, using Zen Blue software for microscope control and data acquisition. Time-lapse movies were acquired using a spinning-disc confocal system (Nikon, Shinjuku City, Tokyo, Japan) mounted on a Zeiss Axio Observer. MV151-labeled 20S proteasome were analyzed in Imaris 7.6 using the manual particle-tracking module. 20S proteasome with velocity higher than 0.1 μm/s within our tracking time-period of 10–20 secs (0.5 s/frame) were considered as moving uni-directionally, whereas those that moved less than 0.1 μm/sec were considered stationary.

*SDD-AGE (Semi-denaturing detergent agarose gel electrophoresis)*—TDP-43 oligomerization or polymerization were assessed as previously described [41–44]. In brief, cells transfescted with indicated plasmids or mouse brain cortex or spinal cord tissue samples were lyzed with Lysis buffer (50 mM Tris–HCl (pH 7.5), 150 mM NaCl, 0.5% NP-40 (IGEPAL), 0.5% Triton X-100 and supplemented with 1× protease inhibitor, 10 mM PMSF and phosphatase inhibitors (5 mM NaF and 5 mM Na_3_VO_4_)_._ After rotating for 30 min at 4 °C, centrifuge it for 20 min at 12,000 rpm in 4 °C. 75 µg of each sample was collected and diluted in 1× SDD-AGE sample buffer (41.25 mM Tris–HCl (pH 6.8), 5% glycerol, 1% SDS, 0.1% bromphenol-blue) and analyzed by SDD-AGE by horizontal 1.5% agarose gel electrophoresis (gel: 1.5% agarose in 1× TAE buffer and 0.1% SDS) in running buffer (1× TGS buffer and 1xTAE buffer) for 20 min at 80 V, and then 210 min at 150 V at 4 °C. Transfer to 0.45 um PVDF membrane in 1× Transfer buffer (25 mM Tris, 192 mM glycine,15% methanol) at 300 V for 240 min at 4 °C. Detection of TDP-43 monomers, oligomers, and high-molecular-weight species was performed by standard Western Blot using anti-TDP-43 antibody.

*Native page and in-gel activity assay*—In brief, mouse brain cortex or spinal cord tissue samples were lyzed with Proteasome assay lysis buffer (20 mM Tris–HCl (pH 7.5), 5 mM MgCl_2_, 1 mM DTT, 2 mM ATP and supplemented with 1× protease inhibitor, 10 mM PMSF and phosphatase inhibitors (5 mM NaF and 5 mM Na_3_VO_4)._ After rotating for 30 min at 4 °C, centrifuge it for 20 min at 12000 rpm in 4 °C. 75 µg of each sample was collected and diluted in 1× Native sample buffer (50 mM Tris–HCl (pH 7.5), 10% glycerol, 0.1% Xylene cyanol) and analyzed by 3–8% Novex Tris–Glycine Page (Thermo Fisher Scientific) in running buffer (90 mM Tris, 90 mM Boric Acid, 5 mM MgCl_2_, 1 mM DTT and 0.5 mM EDTA (pH 7.5)) for 240 min at 130 V, at 4 °C. The gels were subsequently incubated for 30 min at 37 °C in an in-gel activity buffer (20 mM Tris–HCl, 1 mM ATP, 5 mM MgCl₂) supplemented with the proteasome substrate Suc-LLVY-AMC (100 µM) to enable visualization of the resolved proteasome complexes. Following the in-gel activity assay, the gel was transferred to 0.45 um PVDF membrane in 1× Transfer buffer (25 mM Tris, 192 mM Glycine,10% Methanol) at 90 V for 190 min at 4 °C. Detection of 19S, 20S and 26S proteasome subunits levels was performed by standard Western Blot using anti-PSMD4 and anti-PMSA1 antibodies. The intensities of the 19S, 20S, and 26S proteasome bands on native page were normalized to the corresponding α-tubulin loading control on SDS–PAGE. Densitometric analyses were performed using AlphaEase software (Alpha Innotech, San Leandro, CA, USA).

*CFP/YFP förster resonance energy transfer (FRET) microscopy and image analysis*—FRET is a powerful technique for monitoring protein–protein interactions in living cells, where one protein is tagged with a donor fluorophore (e.g., CFP) and the other with an acceptor (e.g., YFP). When the two proteins are within ~50 Å, energy transfer occurs from the excited CFP donor (excitation: 440 nm; emission: 470–500 nm) to the YFP acceptor (excitation: 515 nm; emission: 535–565 nm), generating a FRET emission signal (excitation: 440 nm; emission: 535–565 nm), indicative of close proximity and likely direct interaction. Specifically, fluorescence intensities of the donor CFP, acceptor YFP, and raw FRET channels were measured from the acquired fluorescence images to generate the quantitative values used for FRET analysis. Three separate images were collected to quantify FRET, and the measured values were corrected using the specified FRET correction equation (FRETc). (1) CFP donor images (CFP’) (excitation at 440 nm, emission range between 470 and 500 nm); (2) YFP acceptor images (YFP’) (excitation at 515 nm, emission range between 535 and 565 nm) and (3) FRET images (excitation at 440 nm, emission range between 535 and 565 nm). The equation used here was FRETc = FRET—CFP’—YFP’. The raw FRET images composed of FRET_C_ images and the background resulting from the bleed through of CFP donor (CFP’) and the cross-excitation of YFP acceptor (YFP’). The backgrounds were constant proportion of CFP or YFP fluorescent and can be determined using the cells transfected with either CFP donor or YFP acceptor only and measured through the CFP, YFP and FRET fluorescent sets. The corrected FRETc images were obtained by subtracting background signals of CFP and YFP from the raw FRET images [45, 46]. To evaluate the interaction between TNKS and TDP-43, cells expressing YFP and CFP–TDP-43 were used as a negative control. The FRETc signals of YFP-TNKS/CFP-TDP-43 and YFP-TNKS/CFP-TDP-43-A315T were normalized to the FRETc signal of the YFP/CFP-TDP-43 control to obtain fold-change values. Similarly, to assess the interaction between TNKS and PI31, cells expressing YFP and CFP–PI31 were used as a negative control, and the FRETc signal of YFP-TNKS/CFP-PI31 was normalized to that of the YFP/CFP-PI31 control.

*Statistical analyses*—Statistical analyses were performed using GraphPad Prism software. Data are presented as mean ± SEM. Comparisons between two groups were performed using either unpaired or paired two-tailed Student’s t-tests, as appropriate. Comparisons among more than two groups were performed using one-way ANOVA. For 20S proteasome activity assays (Suc-LLVY-AMC) involving multiple groups across different time points, data were analyzed using two-way ANOVA. The proportions of stationary and moving proteasomes, as well as the proportions of anterograde and retrograde movements, were compared using Fisher's exact test [47].

## Results

### Overexpression of TDP-43 results in the formation of insoluble oligomers, polymers, and aggregates, reduces proteasome activity, and consequently causes the accumulation of ubiquitinated proteins

**Fig. 1:**
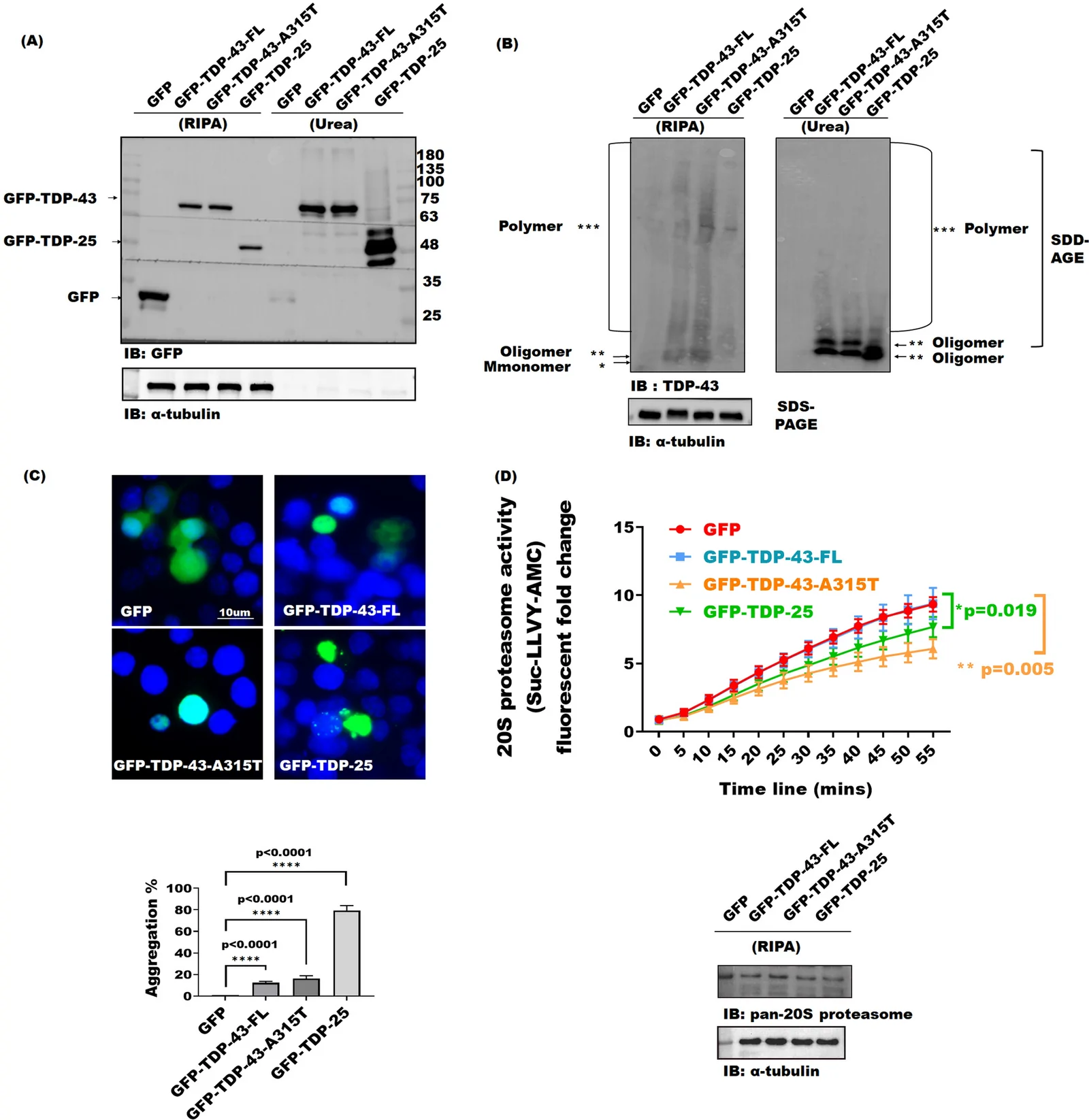
Overexpression of TDP-43 forms insoluble oligomers, polymers and aggregates, leading to accumulation of ubiquitinated proteins and decreases 20S proteasome activity. **A** Relative insolubilities patterns of GFP–TDP-43-FL, TDP-43-A315T mutant and TDP-25 protein species. N2a cells transfected with indicated plasmids were separated into RIPA- and urea fractions as described in the Materials and Methods (N = 3 independent biological replicates). **B** SDD-AGE and immunoblotting were performed using equal amounts of cell lysates proteins to analyse the oligomerization and aggregation patterns of GFP–TDP-43-FL, the TDP-43-A315T mutant, and TDP-25. All blots were probed with a TDP-43 antibody (*monomers; **oligomers; ***polymers). In parallel, immunoblotting of α-tubulin was used as a loading control to confirm comparable protein input across different transfected conditions (N = 3 independent biological replicates). **C** Immunofluorescence revealed the nuclear and cytosolic distribution and aggregation patterns of GFP–TDP-43-FL, the A315T mutant, and TDP-25 protein species. Quantification of the percentage of cells containing aggregates in N2a cells transfected with GFP or GFP–TDP-43 constructs was presented as a bar graph. Data were presented as the mean ± SEM from three independent cell-culture experiments (N = 3 biological replicates), with a total of 50 cells analysed per condition (n = 50 technical measurements). Statistical analyses were performed using pooled technical measurements. Groups were compared using one-way ANOVA. **D** 20S proteasome activity of cell lysates of N2a cells transfected with indicated plasmids were measured with 20S proteasome specific substrate fluorogenic peptides, Suc-Leu-Leu-Val-Tyr-AMC (Suc-LLVY-AMC). Time-dependent changes in 20S proteasome activity were presented as line graphs. Data are shown as the mean ± SEM from at least three independent experiments (N ≥ 3 independent biological replicates). Statistical significance was analyzed using two-way ANOVA to compare groups across multiple time points. In parallel, immunoblotting for 20S proteasome and α-tubulin was performed to confirm comparable 20S proteasome expression levels and equal protein loading among the different transfection conditions

Although endogenous TDP-43 is tightly autoregulated, multiple independent clinical studies have reported that a 1.2–1.5-fold increase in total TDP-43 protein is observed in the brain tissue, plasma, and cerebrospinal fluid of patients with ALS and FTLD compared to healthy controls [48–52]. In ALS and related TDP-43 proteinopathies, this slight increase in TDP-43 protein is sufficient to promote its pathological misfolding, hyperphosphorylation and ubiquitination, leading to formation of oligomers or aggregates predominantly detected in the RIPA-insoluble/urea-soluble fractions rather than the normally folded protein existing in the RIPA soluble pool, thereby impairing protein homeostasis [5, 37, 38, 53, 54]. Accordingly, here we overexpressed TDP-43 to mimic the disease state of TDP-43-related ALS neuropathy and examined the impact of TDP-43 overexpression on proteasome activity. We transfected equal amounts of GFP control, GFP-tagged human wild-type TDP-43 full-length (TDP-43-FL), TDP-43-A315T, or truncated TDP-25 plasmids into equal numbers of N2a cells. Soluble and insoluble protein fractions were isolated using RIPA and urea buffers, respectively (Fig. 1A). Oliogmeric or polymeric TDP-43 species were then analyzed by semi-denaturing detergent agarose gel electrophoresis (SDD-AGE) (Fig. 1B), while subcellular localization and aggregates formation were examined by immunofluorescence (Fig. 1C). With comparable total TDP-43 protein levels, including both soluble and insoluble fractions, among GFP, GFP–TDP-43-FL, GFP–TDP-43-A315T, and GFP–TDP-25 expressing cells (Fig. S1B (Additional file 1)), these GFP–TDP-43 constructs exhibited distinct solubility profiles and aggregation patterns. GFP proteins were detected exclusively in the RIPA-soluble fraction (Fig. 1A and S1A (Additional file 1)) and displayed diffuse nuclear and cytoplasmic localization without detectable oligomeric or polymeric species (Fig. 1B, C), suggesting that overexpressed GFP remained properly folded and soluble. In contrast, wild-type GFP-TDP-43-FL and mutant GFP–TDP-43-A315T predominantly accumulated in the urea-insoluble fraction (Fig. 1A and S1A (Additional file 1)) as oligomeric species (Fig. 1B), while remaining mainly nuclear without prominent cytoplasmic inclusions or aggregates (Fig. 1C). GFP–TDP-25 showed a distinct pattern, with reduced levels in the RIPA-soluble fraction and predominant accumulation as insoluble oligomeric species in the urea fraction (Fig. 1A, B, and S1A (Additional file 1)), accompanied by prominent perinuclear and cytoplasmic punctate aggregates (Fig. 1C). These findings indicate that GFP–TDP-43-FL and GFP–TDP-43-A315T preferentially form insoluble oligomers without significant cytoplasmic aggregation, whereas GFP–TDP-25 exhibits a stronger aggregation propensity, forming both insoluble oligomers and prominent cytoplasmic aggregates [36, 37].

To further assess the effect of these TDP-43 species in endogenous 20S proteasome activity, equal amounts of protein from the transfected cell lysates were incubated with the specific 20S proteasome fluorogenic substrate Suc-LLVY-AMC. The levels of 20S proteasome and α-tubulin were comparable among the experimental groups, excluding differences in proteasome abundance or protein loading as confounding factors (Fig. 1D). In 20S proteasome activity assay, similar to earlier findings [55, 56], endogenous 20S proteasome activity was significantly inhibited, especially in cells transfected with GFP-TDP-43-A315T mutant and the truncated GFP-TDP-25 pathological TDP-43 proteins (Fig. 1D and S1C (Additional file 1)). To further confirm the impact of TDP-43 overexpression on proteasome activity, we then transfected NSC34 cells with TDP-FL and truncated TDP-25 fragments and employed a GFP-tagged ubiquitin mutant substrate (Ub^G76V−^GFP) to assess proteasome activity. The Gly76-to-Val mutation prevents deubiquitination and causes continuous polyubiquitination of GFP, which serves as a signal for targeting the GFP protein to the 26S proteasome for degradation [57]. The expression level of Ub^G76V^-GFP is inversely correlated with intracellular 26S proteasome activity. As shown in Fig. S1D (Additional file 1), Ub^G76V^-GFP levels were significantly increased in NSC34 cells overexpressing TDP-43-FL or TDP-25 compared with control cells, with the most pronounced accumulation observed in TDP-25-expressing cells. Those treated with proteasome inhibitor MG132 are as positive control which show abundant Ub^G76V^-GFP proteins accumulation (Fig. S1D (Additional file 1)). Furthermore, consistent with proteasome dysfunction, cells expressing GFP–TDP-43-FL, GFP–TDP-43-A315T, or GFP–TDP-25 exhibited a significant increase of endogenous polyubiquitylated protein levels compared to GFP control group, (Fig. S1E (Additional file 1)). These results are consistent with the possibility that overexpressed TDP-43 species with varying solubility, whether existing as insoluble oligomers only or forming cytoplasmic aggregates, impair proteasome activity and thereby contribute to the accumulation of polyubiquitinated proteins and disrupting of cellular protein homeostasis, highlighting the pathogenic significance of oligomeric TDP-43 species in disease progression [58, 59].

### Pathological TDP-43 reduces 20S proteasome activity in both cellular and Prp-TDP-43^A315T^ Tg mouse models

**Fig. 2:**
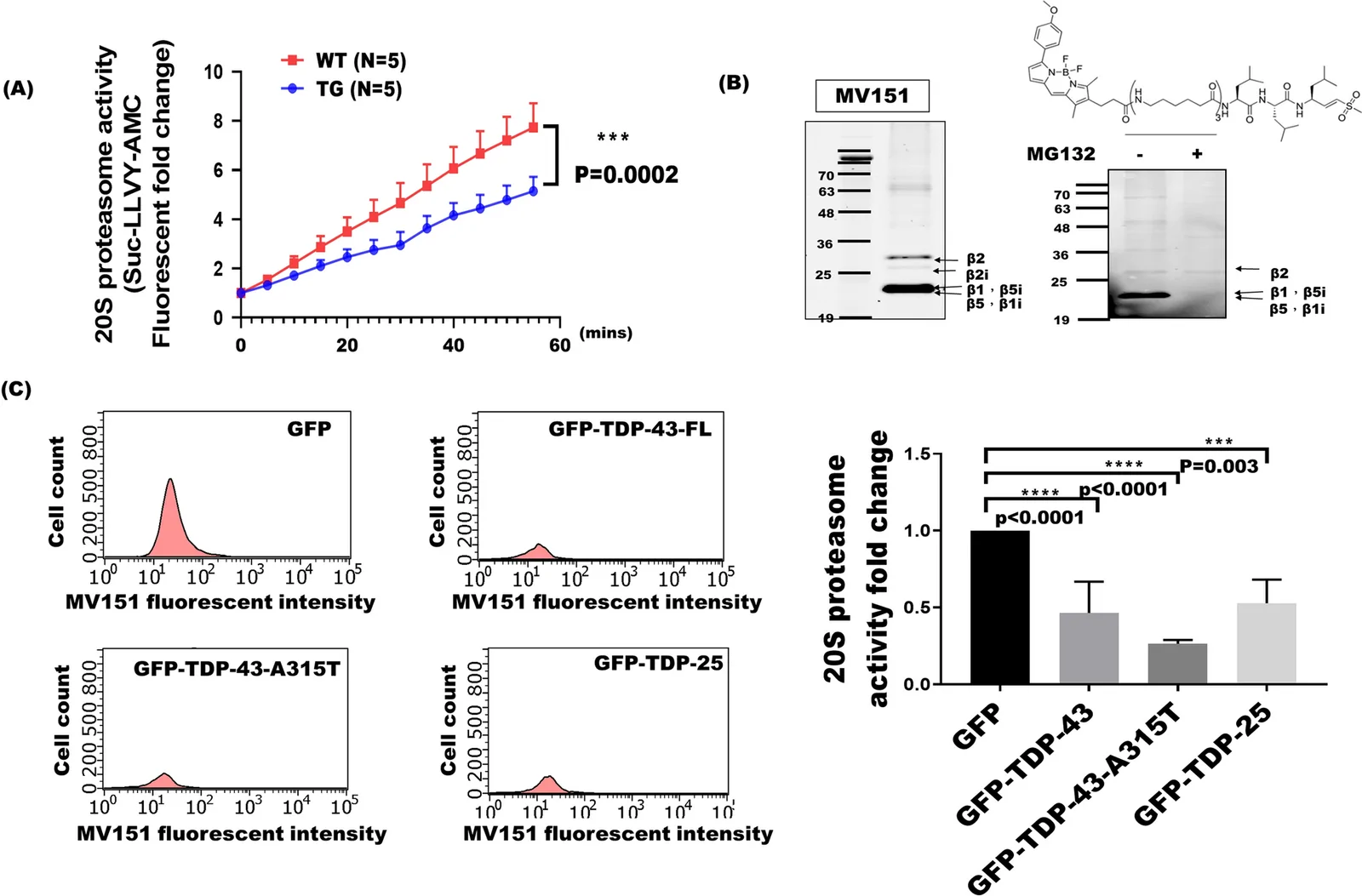
Pathological TDP-43 reduces 20S proteasome activity in both cellular and Prp-TDP-43^A315T^ Tg mouse models. **A** 20S proteasome activity of equal amount of tissue lysates proteins derived from non-Tg wild-type littermate controls (N = 5 mice) and Prp-TDP-43^A315T^ mice (N = 5 mice) spinal cord were measured with 20S proteasome specific substrate fluorogenic peptides Suc-LLVY-AMC. Time-dependent changes in 20S proteasome activity were presented as line graphs. Data were presented as the mean ± SEM from five independent biological replicates per condition (N = 5 mice). Comparisons between two groups were performed using a paired two-tailed Student's t-test. In parallel, immunoblotting for 20S proteasome and α-tubulin was performed to confirm comparable 20S proteasome expression levels and equal protein loading among non-Tg wild-type littermate controls and Prp-TDP-43^A315T^ mice (Fig. S2A (Additional file 1). **B** In-gel MV151 labeling was performed by incubating cell lysates proteins from N2a cells with 1 μM MV151 for 1 h at 37 °C, followed by SDS-PAGE analysis. MV151-labeled catalytic β1, β2, and β5 subunits of active 20S proteasomes were detected as fluorescent signals on the gel, whereas no fluorescent signal was detected in samples treated with the 20S proteasome inhibitor MG132 (N = 3 independent biological replicates) (see Materials and Methods). **C** The fluorescence intensity of MV151-labeled 20S proteasomes was further analyzed by flow cytometry. Representative one-dimensional histograms showing MV151 fluorescence intensity (X-axis) versus cell number (Y-axis) in N2a cells transfected with GFP or GFP–TDP-43 constructs were presented. A total of 5,000 GFP-positive cells were sorted from each sample. Relative 20S proteasome activity was calculated by multiplying the mean MV151 fluorescence intensity by the number of MV151-positive cells. The 20S proteasome activity of GFP-transfected cells was set to 1, and fold changes in 20S proteasome activity in cells expressing the indicated GFP–TDP-43 constructs were determined and presented as bar graphs. Data were presented as the mean ± SEM from three independent cell-culture experiments (N = 3 biological replicates), with a total of 5000 cells analysed per condition (n = 5000 technical measurements). Statistical analyses were performed using pooled technical measurements. Groups were compared using one-way ANOVA)

To verify the effect of TDP-43 on proteasome in cell culture model, we assessed endogenous 20S proteasome activity in spinal cords from non-Tg wild-type control mice and ALS model Prp-TDP-43^A315T^ Tg mice (Fig. 2B and Fig. S2C (Additional file 1)). The Prp-TDP-43^A315T^ Tg line expresses Flag-tagged human TDP-43-A315T (Flag-hTDP-43-A315T), a mutation associated with familial ALS, under the control of the mouse Prp promoter (see Materials and Methods). Hemizygous mice exhibit a progressive and fatal neurodegenerative phenotype resembling both ALS and FTLD-U, and develop pathological insoluble ubiquitinated proteins in specific neuronal populations, including frontal cortex layer V pyramidal neurons and spinal motor neurons, accompanied by activation of local astrocytes and microglia [33, 60, 61]. They also display a progressive motor phenotype characterized by arched back, lower limb paralysis, gait abnormalities, denervation at neuromuscular junctions, gastrocnemius muscle atrophy, hind-limb clasping, and loss of both upper and lower motor axons, particularly large-caliber axons [33, 35, 54]. We found that, despite TDP-43 aggregates were not observed in Prp-TDP-43^A315T^ Tg spinal cord [33, 54, 62], typical pathological hallmark phosphorylated TDP-43 oligomers and polymers were significantly detected in Prp-TDP-43^A315T^ Tg mice during disease progression, with the highest levels observed at late stages, suggesting that the pathological TDP-43-A315T mutant majorly express as the form of phosphorylated oligomers (Fig. S2B and S2C (Additional file 1))[54]. Consistent with our findings in the cell model (Fig. 1D), 20S proteasome activity was significantly reduced in Prp-TDP-43^A315T^ Tg mice compared with wild-type littermates, with comparable expression levels of the 20S proteasome and the loading control α-tubulin between groups (Fig. 2A; Fig. S2A (Additional file 1)). We further analyzed 20S proteasome activity using the activity-based fluorescent probe MV151. MV151 specifically labels the catalytic β1, β2, and β5 subunits of active 20S proteasomes, and the MV151-labeled β1, β2, and β5 fluorescent signal were visualized and detected by in-gel fluorescence scanning, while no signal was observed in samples treated with the 20S proteasome inhibitor MG132 (Fig. 2B). Compared with GFP control group, MV151-labeled 20S proteasome activity was significantly reduced in cells overexpressing GFP–TDP-43, GFP-TDP-43-A315T or GFP–TDP-25 (Fig. 2C and Fig. S2D (Additional file 1)). Taken together, our findings indicate that oligomeric TDP-43 species may contribute to proteasome dysfunction and the impaired cellular protein homeostasis in both TDP-43-overexpressing cell models and Prp-TDP-43^A315T^ transgenic mice.

### Overexpression of pathological TDP-43 interrupts the axonal transport of 20S proteasome

**Fig. 3:**
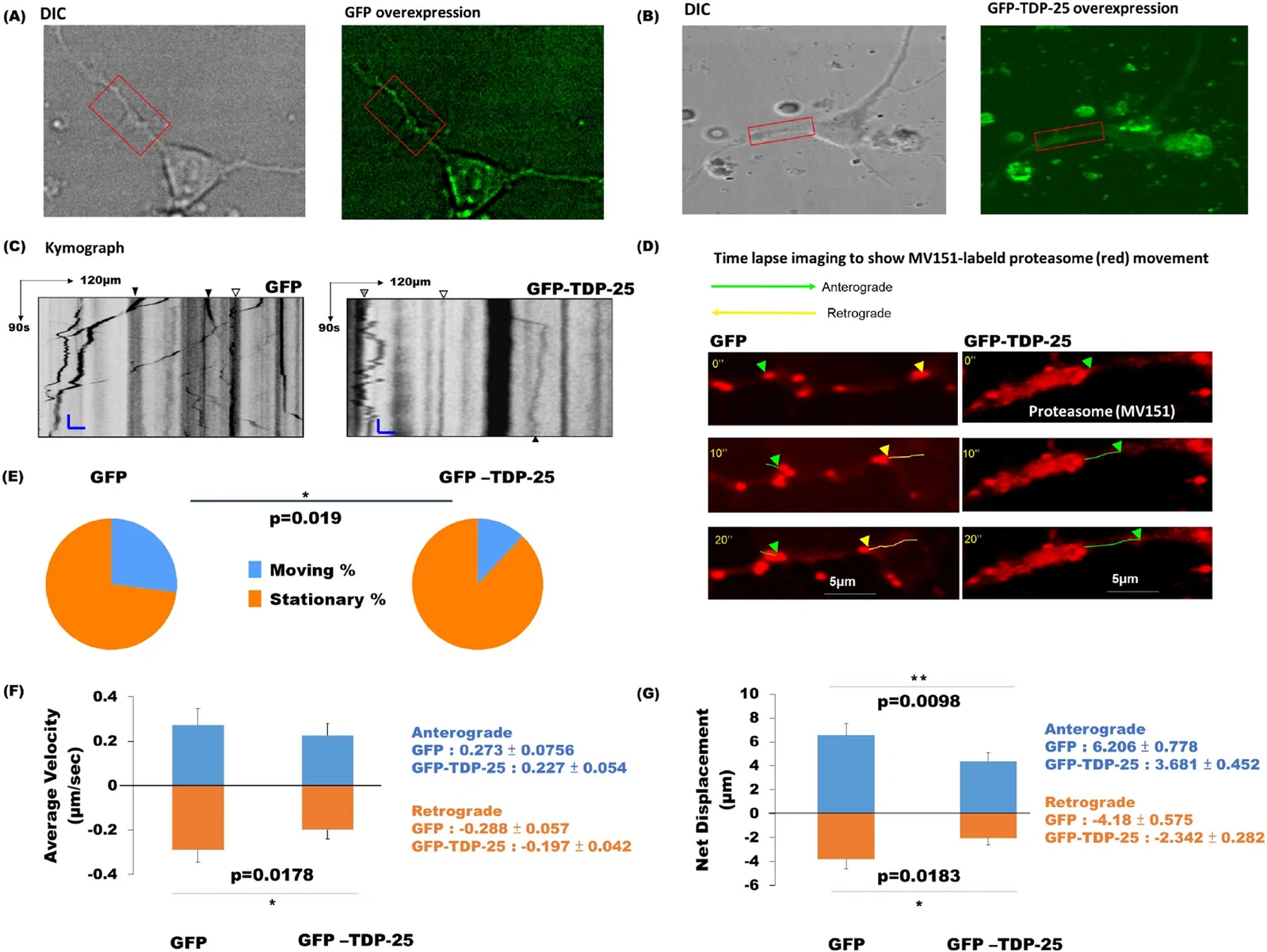
Overexpression of pathological truncated GFP-TDP-25 interrupts the axonal transport of 20S proteasome in wild-type primary cortex neuron. **A**, **B** Primary cortical neurons derived from wild-type E16.5 mouse brains were transfected at DIV5 with GFP or GFP–TDP-25 for 48 h, and live-cell imaging was performed at DIV7 to examine the effects of pathological truncated GFP–TDP-25 on axonal proteasome movement. **C** Kymographs tracing axonal proteasomes in live primary cortical neurons. X-axis indicates the axon length of 120 µm; Y-axis represents a time period of 90 s. **D** Representative images showed the movement of 20S proteasomes (red spots) in primary cortical neurons over time. Yellow and green arrows indicate retrograde and anterograde proteasomes, respectively, and the corresponding tracing lines depict their trajectories over time. **E** 20S proteasomes with velocity higher than 0.1 µm/sec within a 10–20 s tracking time-period (0.5 s/frame) were defined as moving in a single direction, while those moving slower than 0.1 µm/sec were considered as stationary. The pie charts showed the proportions (%) of stationary versus uni- or bi-directionally moving 20S proteasomes. Data were obtained from three independent biological replicates (N = 3 mice) with 15 technical measurements per condition (n = 15 individual 20S proteasome granules). Statistical analyses were performed using pooled technical measurements. The proportions of stationary and moving proteasomes was compared using Fisher's exact test. **F**, **G** Bar diagram represented the average anterograde and retrograde displacements (μm) and velocities (μm/s) of proteasomes. Data were presented as the mean ± SEM from three independent biological replicates (N = 3 mice) with 9–13 technical measurements per condition (n = 9 – 13 individual 20S proteasome granules). Statistical analyses were performed using pooled technical measurements. Comparisons between groups were performed using an unpaired two-tailed Student’s t-test

Neurons possess a highly polarized morphology, with long axons extending far from the cell body. In addition to 20S proteasome activity, maintaining local protein homeostasis particularly depends on the proper transport of proteasomes along the axon to cellular compartments where local protein turnover is required. Disruptions in this transport not only impair local protein degradation but also promote the accumulation of ubiquitinated protein aggregates. We found that TDP-43 overexpression attenuates 20S proteasome activity. However, whether TDP-43 pathology also disrupts proteasome transport along the axon, further impairing neuronal local protein homeostasis, remains to be investigated. To validate the effect of overexpressed pathological TDP-43 on axonal transport of 20S proteasome, we evaluated the live fluorescent imaging of MV151-labeled 20S proteasome subunit in the primary cortex neuron derived from wild-type mice (Fig. 3). The E16.5 primary cortex neurons were transfected with GFP- or truncated GFP-TDP-25 and the endogenous 20S proteasome was labeled with MV151. We used time-lapse imaging for live-cell fluorescence to study the movement of endogenous MV151-labeled 20S proteasome within axon of DIV7 primary cortex neurons. Live-cell fluorescence imaging of mouse cortical neurons revealed GFP and punctate-like aggregates of GFP-TDP-25 proteins throughout the soma and axon (Fig. 3A, B). Kymograph shows stationary (white arrowhead), oscillatory (gray arrowhead), and motile (black arrowhead) 20S proteasome (120 μm and 90 s) (Fig. 3C). Time lapse imaging showed MV151-labeled endogenous 20S proteasome anterograde and retrograde movements within axon (Fig. 3D). The percentage of moving 20S proteasome was significantly reduced in primary cortical neurons transfected with GFP–TDP-25 compared with GFP controls, indicating that overexpression of pathological TDP-25 impairs 20S proteasome transport within axons (Fig. 3E). In addition, the MV151-labeled 20S proteasome in GFP-TDP-25-transfected primary cortex neurons showed significant decrease in average velocity and net displacement as compared with GFP controls (Fig. 3F, G) (Movie A for GFP and Movie B for GFP-TDP-25). A comparable impairment in MV151-labeled 20S proteasome motility was also observed in primary cortical neurons expressing GFP–TDP-43-FL (Fig. S3 (Additional file 1)) (Movies C for GFP-TDP-FL), indicating that excessive accumulation of both truncated TDP-25 and full-length TDP-43 disrupt proteasome transport along axons.

### Pathological TDP-43 sequesters TNKS into insoluble fraction, and further attenuates TNKS–PI31 interaction, PI31 ADP-ribosylation and impairs 19S/20S proteasome assembly

**Fig. 4:**
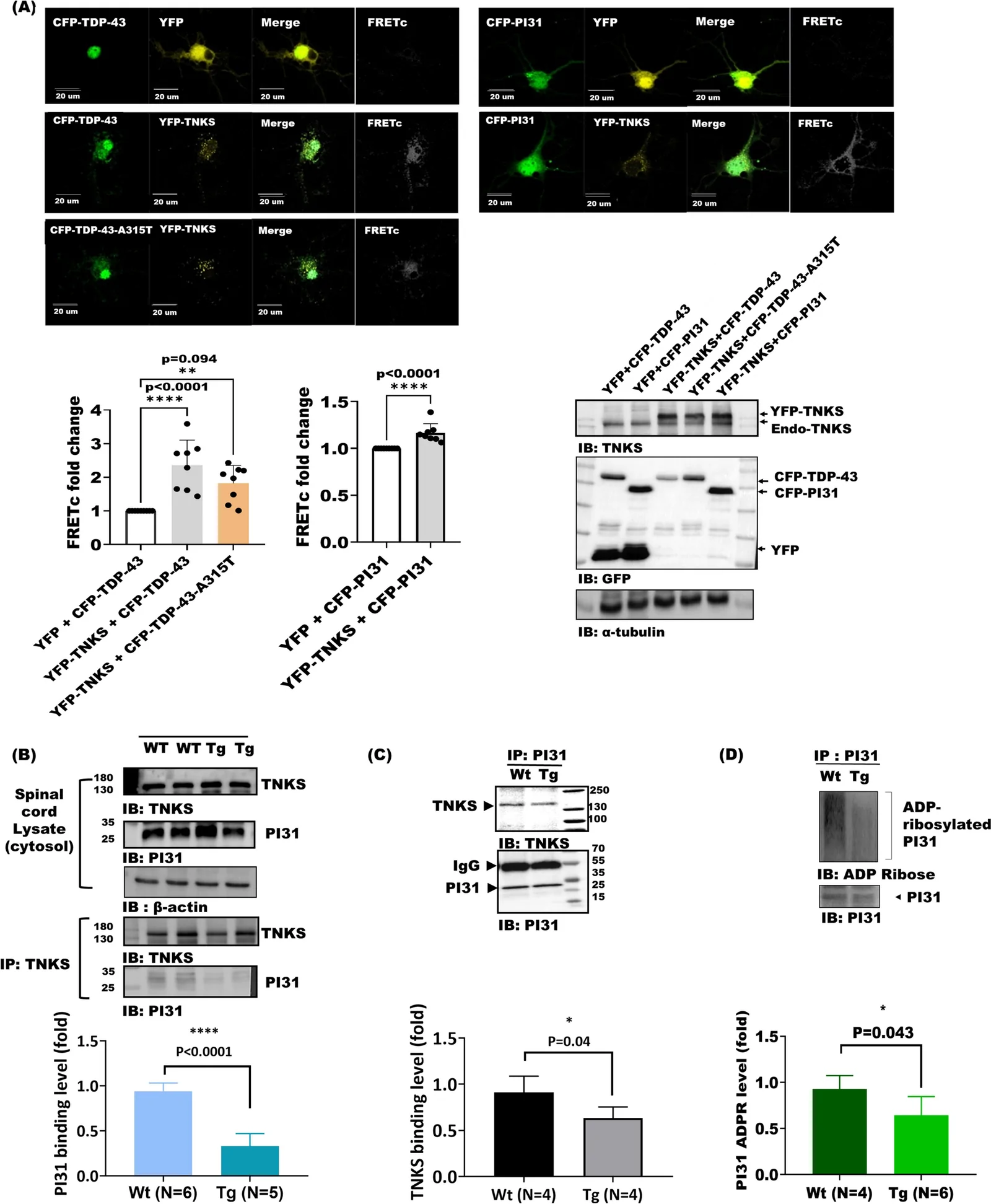
TNKS–PI31 interaction and PI31 ADP-ribosylation are attenuated in Prp-TDP-43^A315T^ mice. **A** Direct interaction between YFP-TNKS and CFP-TDP-43 or CFP-PI31. Primary hippocampal neurons were transfected with indicated plasmids for 48 h. Data were presented as the mean ± SEM from three independent biological replicates (N = 3 mice) with 8–9 technical measurements per condition (n = 8 – 9 cells). Statistical analyses were performed using pooled technical measurements. Bar graphs showed the fold change in FRETc signals. FRETc signals fold change were significantly increased in cells co-expressing YFP-TNKS and CFP-TDP-43 (2.3556 ± 0.25) and YFP-TNKS and CFP-TDP-43-A315T (1.821 ± 0.25) compared with their negative control groups, YFP and CFP-TDP-43. The positive control group, YFP-TNKS and CFP-PI31, also exhibited a significant increase (1.165 ± 0.16) compared with their corresponding negative control group (YFP and CFP-PI31). Statistical analyses were performed using ordinary one-way ANOVA for the TNKS/TDP-43 groups and an unpaired two-tailed Student’s t-test for the TNKS/PI31 groups. Immunoblot analysis confirmed comparable expression levels of the indicated proteins among all groups. **B**, **C** Cytosolic lysates prepared from the spinal cord cervical part of non-Tg wild-type and Prp-TDP-43^A315T^ mice were immunoprecipitated with TNKS (**B**) or PI31 (**C**) antibody and immunoblotted with anti-TNKS and anti-PI31 antibodies. Western blotting and bar graphs showed lower interaction levels between PI31 and TNKS in Prp-TDP-43^A315T^ mice comparing to those of non-Tg wild-type. PI31 levels were normalized to TNKS in (**B**); TNKS levels were normalized to PI31 in (**C**), and two groups were compared using unpaired t-test, two-tailed p value. **D** As addressed in (**B**, **C**), cytosolic lysates prepared from the spinal cord cervical tissues were immunoprecipitated with PI31 antibody and immunoblotted with anti-ADP-ribose antibodies. Western blotting and bar graph showed relatively lower ADP-ribosylation levels of PI31 in Prp-TDP-43^A315T^ mice comparing to those of non-Tg wild-type. PI31 ADPR levels were normalized to PI31, and two groups were compared using unpaired t-test, two-tailed *p* value. Data were presented as the mean ± SEM from 4 to 6 biological replicates per condition (N = 4–6 mice)

**Fig. 5:**
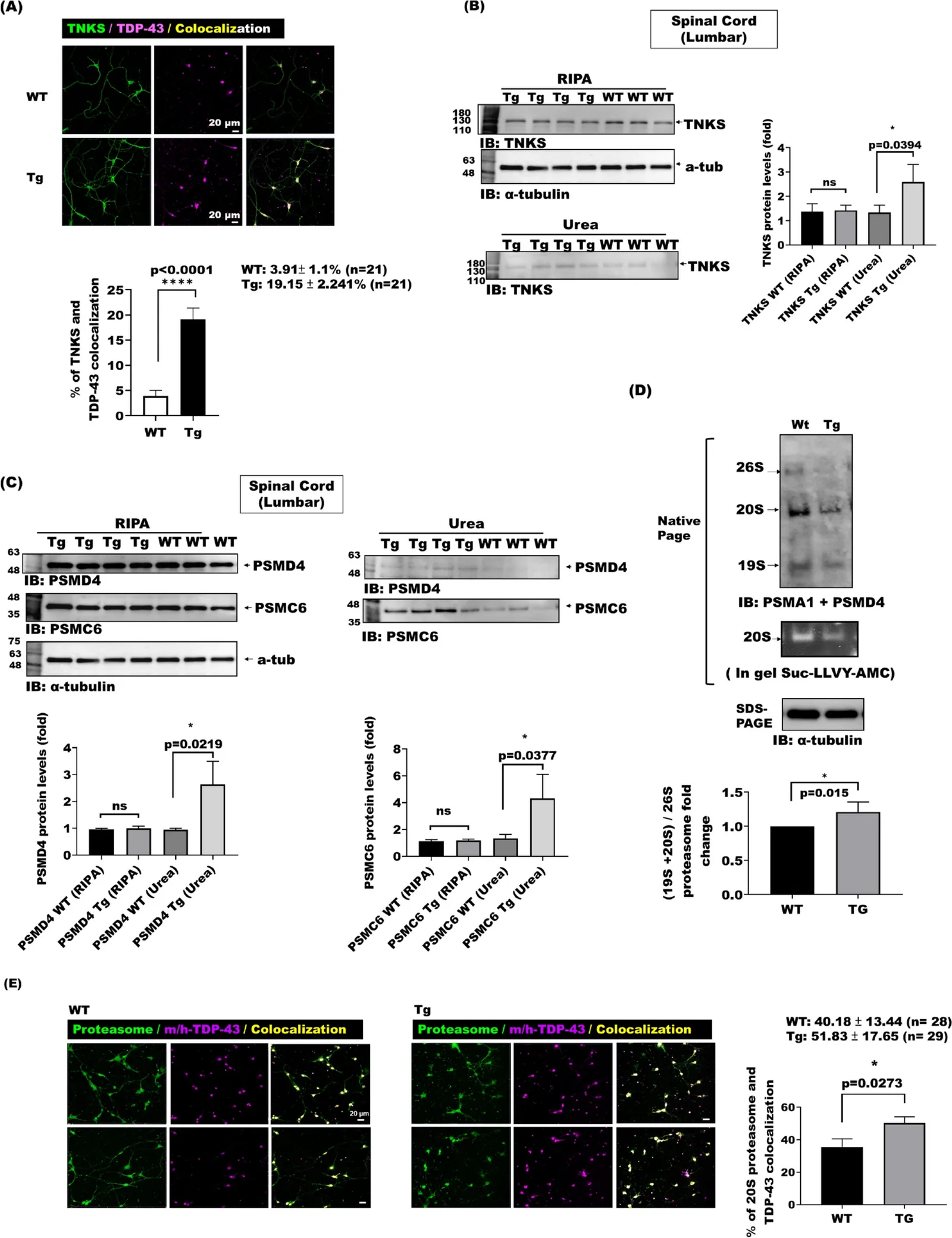
TDP-43 sequesters TNKS into insoluble fraction and ultimately impairing 19S/20S proteasome assembly. **A** Primary motor neurons derived from spinal cord tissues of non-Tg wild-type and Prp-TDP-43^A315T^ mice were stained with anti-TNKS (green) and anti-TDP-43 (magenta) antibodies. The % of TDP-43 colocalized with TNKS between non-Tg wild-type and Prp-TDP-43^A315T^ mice were shown in the bar graph. Data were presented as the mean ± SEM from three independent biological replicates (N = 3 mice) with 21 technical measurements per condition (n = 21 cells). Statistical analyses were performed using pooled technical measurements. Independent two groups were compared using *t*-test, two-tailed *p* value. **B**, **C** Western blotting and bar graph showed the comparison of the soluble and insoluble TNKS (**B**) and 19S proteasome (PSMD4 and PSMC6) (**C**) protein levels in RIPA and urea fraction extracted from spinal cord lumber tissues of non-Tg wild-type littermate controls (N = 3 mice) and Prp-TDP-43^A315T^ mice (N = 4 mice). Data were presented as the mean ± SEM from 3–4 biological replicates per condition (N = 3–4 mice). Independent two groups were compared using unpaired t-test, two-tailed *p* value. **D** The assembly status of 26S proteasome complexes was analyzed by native gel electrophoresis using spinal cord tissue lysates from non-transgenic wild-type controls (N = 5 mice) and Prp-TDP-43^A315T^ mice (N = 5 mice). 19S, 20S and 26S proteasome species were detected by immunoblotting with antibodies against PSMA1 and PSMD4, allowing visualization of disassembled 19S and 20S proteasome subcomplexes as well as intact 26S proteasome complexes. The 20S proteasome activity was further analyzed by in-gel 20S proteasome activity assay using the chymotrypsin-like substrate Suc-LLVY-AMC (In gel Suc-LLVY-AMC). In parallel, immunoblotting for internal control α-tubulin was performed to confirm equal protein loading among non-Tg wild-type littermate controls and Prp-TDP-43^A315T^ mice. Native gel revealed a reduction in levels of intact 19S, 20S, and 26S proteasome complexes. The intensities of the 19S, 20S, and 26S proteasome bands detected by native PAGE were normalized to the corresponding α-tubulin loading control measured by SDS–PAGE. The bar graph shows the fold change in the ratio of disassembled 19S and 20S proteasome subcomplexes to intact 26S proteasome complexes. Data were presented as the mean ± SEM from 5 biological replicates per condition (N = 5 mice). Independent two groups were compared using unpaired t-test, two-tailed *p* value. **E** Primary motor neurons derived from spinal cord tissues of non-Tg wild-type and Prp-TDP-43^A315T^ mice were stained with MV151-labeled 20S proteasome (green) and anti-TDP-43 (m/h-TDP-43) (magenta) antibody. The % of TDP-43 colocalized with 20S proteasome between non-Tg wild-type and Prp-TDP-43^A315T^ Tg mice were shown in the bar graph (Biological repeats, N = 3 mice, technical measurements, n = 28–29 cells). Statistical analyses were performed using pooled technical measurements. Independent two groups were compared using unpaired t-test, two-tailed p value

Our findings demonstrated that pathological TDP-43 impairs both proteasome activation (Figs. 1 and 2) and proteasome axonal transport (Fig. 3). These observations prompted us to explore the underlying molecular mechanism. Given that TNKS–PI31 interaction and TNKS-mediated PI31 ADP-ribosylation are required for 19S–20S proteasome assembly and subsequent 26S proteasome activation [22], we further investigated whether TDP-43 disrupts proteasome function by interfering with the TNKS-PI31-ADP ribosylation signaling pathway. Previous studies have demonstrated interactions between TNKS and PI31 in drosophila embryos and 293 cells [19]. In addition to PI31, TDP-43 has also been identified as a binding partner of TNKS through a canonical TNKS-binding motif (RxxΦDG) located in the RRM1 domain of TDP-43 [60, 61]. To examine whether TNKS also associates with both TDP-43 and PI31 in neurons, we performed FRET assays to examine the protein–protein interaction patterns of PI31/TNKS and TDP-43/TNKS in primary hippocampal neurons. Compared with the YFP/CFP-TDP-43 negative control, both YFP-TNKS/CFP-TDP-43 and YFP-TNKS/CFP-TDP-43-A315T exhibited prominent FRETc signals in the cytoplasm and increased FRETc fold changes (Fig. 4A), indicating that TNKS predominantly interacts with both wild-type TDP-43 and TDP-43-A315T mutant in the cytoplasm of neurons. Similarly, compared with the YFP/CFP-PI31 negative control, YFP-TNKS/CFP-PI31 exhibited strong cytoplasmic FRETc signals and a significantly increased FRETc fold change (Fig. 4A), indicating that TNKS also interacts with PI31 in the cytoplasm of neurons. Expression levels of the YFP- and CFP-fusion proteins were comparable across the experimental groups. These results demonstrated that TNKS interacts with both TDP-43 and PI31 in neurons. We next examined whether pathological TDP-43 interferes with TNKS-PI31 interaction, and whether this alteration is associated with reduced TNKS-mediated PI31 ADP-ribosylation. We immunoprecipitated TNKS or PI31 in non-Tg wild-type control and Prp-TDP-43^A315T^ Tg mice spinal cord tissue lysates and found that TNKS-PI31 association level was lower in Prp-TDP-43^A315T^ Tg mice than that in non-Tg wild-type control mice (Fig. 4B, C). Consistently, PI31 ADP-ribosylation levels were also attenuated in Prp-TDP-43^A315T^ Tg mice as compared to that of wild-type mice (Fig. 4D). These results demonstrate that pathological TDP-43 is associated with reduced TNKS-PI31 interaction and TNKS-mediated PI31 ADP-ribosylation. We next compared the colocalization pattern of TNKS and TDP-43 in non-transgenic (non-Tg) wild-type and Prp-TDP-43^A315T^ transgenic mice to assess the effect of pathological TDP-43 on their spatial distribution. We found a significant increase in the percentage of endogenous TNKS colocalized with pathological TDP-43 in Prp-TDP-43^A315T^ mice compared with non-Tg controls (Fig. 5A). This finding suggests that pathological TDP-43 captures and isolates TNKS in the same cellular compartments. Since excessive pathological TDP-43 is prone to form insoluble oligomers or aggregates [54], we next examined whether pathological TDP-43 sequesters TNKS into the insoluble fraction, which could potentially interfering with TNKS–PI31 signaling cascade. Spinal cord tissue lysate from non-Tg wild-type control mice and Prp-TDP-43^A315T^ Tg mice were fractionated into a RIPA-soluble fraction and a urea-extracted insoluble fraction. We found the levels of insoluble TNKS and 19S proteasome subunits, including PSMC6 and PSMD4, in the urea fraction were significantly higher in Prp-TDP-43^A315T^ Tg mice than in non-Tg wild-type control mice (Fig. 5B, C), suggesting that pathological TDP-43 sequesters not only TNKS but also proteasomes subunits into insoluble fraction. Given that TNKS-mediated ADP-ribosylation of PI31 enhances the 26S proteasome assembly from 19 and 20S proteasomes [22], we next assessed the impact of pathological TDP-43 on 26S proteasome assembly by determining the ratio of disassembled 19S and 20S proteasome particles to intact 26S proteasome complexes in a native PAGE assay (Fig. 5D). The intensities of the 19S, 20S, and 26S proteasome bands detected by native page were normalized to the corresponding α-tubulin loading control measured by SDS–PAGE. In Prp-TDP-43^A315T^ Tg mice, compared with wild-type controls, the levels of structurally intact and native 26S, 20S, and 19S proteasomes complexes were reduced and increased ratio of 19S and 20S proteasome subunits to 26S proteasome complexes was observed (Fig. 5D). Moreover, in gel Suc-LLVY-AMC assay showed attenuated 20S proteasome activity in Prp-TDP-43^A315T^ Tg mice compared with non-Tg wild-type controls (Fig. 5D). These findings are consistent with the increased accumulation of insoluble proteasome subunits in the urea fraction of Prp-TDP-43^A315T^ Tg mice compared with non-Tg wild-type control mice (Fig. 5C and Fig. S2B–C (Additional file 1)), as well as the increased colocalization of TDP-43 with MV151-labeled 20S proteasomes in motor neurons of Prp-TDP-43^A315T^ Tg mice (Fig. 5E), supporting the notion that pathological TDP-43 sequesters and isolates proteasome subunits into insoluble fractions, thereby contributing to the impaired proteasome complex integrity and assembly. Collectively, our findings suggest that pathological TDP-43 sequesters TNKS and a subset of proteasome components into insoluble fractions, thereby reducing TNKS availability for interaction with PI31 and limiting the availability of soluble 19S and 20S subcomplexes for 26S proteasome assembly.

### TDP-43-TNKS interaction contributes to TNKS/PI31 axis disruption and proteasome dysfunction

**Fig. 6:**
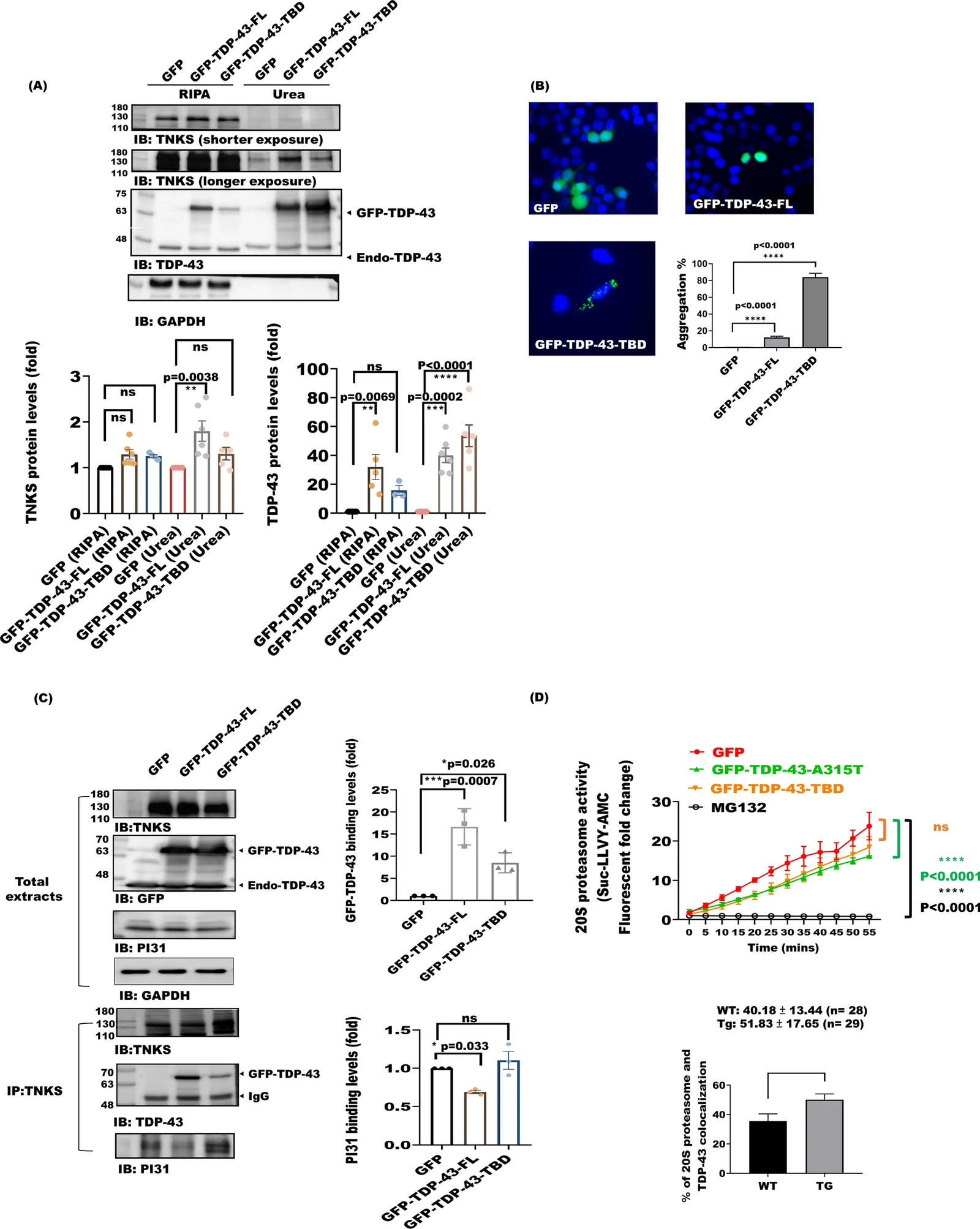
Disruption of the TDP-43–TNKS interaction attenuates TDP-43-induced TNKS sequestration and restores TNKS/PI31 interaction and 20S proteasome activity. TDP-43–TNKS interaction is associated with TNKS/PI31 dysregulation and proteasome dysfunction. **A** Relative insolubilities patterns of GFP, GFP–TDP-43-FL, and GFP–TDP-43-TBD mutant protein species. NSC34 cells transfected with indicated plasmids were separated into RIPA- and urea fractions as described in the Materials and Methods. Western blotting and bar graph showed the comparison of the soluble and insoluble TNKS and GFP-TDP-43 protein levels in RIPA and urea fraction extracted. Data were presented as the mean ± SEM from three independent cell-culture experiments (N = 4–6 biological replicates). Groups were compared using one-way ANOVA. **B** NSC34 cell were transfected with GFP, GFP–TDP-43-FL, and GFP–TDP-43-TBD mutant plasmids. Immunofluorescence and bar graph revealed the nuclear and cytosolic distribution and aggregation patterns of GFP–TDP-43-FL, and GFP–TDP-43-TBD mutant protein species. Data were presented as the mean ± SEM from three independent cell-culture experiments (N = 3 biological replicates), with a total of 50 cells analysed per condition (n = 50 technical measurements). Statistical analyses were performed using pooled technical measurements. Groups were compared using one-way ANOVA. **C** Cytosolic lysates of NSC34 cells transfected with GFP, GFP–TDP-43-FL, and GFP–TDP-43-TBD mutant plasmids were immunoprecipitated with TNKS antibody and immunoblotted with anti-TNKS, TDP-43 and PI31 antibodies. Western blotting and bar graphs showed lower TNKS/GFP-TDP-43-TBD interaction and higher TNKS/PI31 interaction in GFP–TDP-43-TBD-transfected groups compared to GFP–TDP-43 -transfected groups. Pull downed GFP–TDP-43 and PI31 levels were normalized to TNKS. Groups were compared using one-way ANOVA. Data were presented as the mean ± SEM from three independent cell-culture experiments (N = 3 biological replicates). **D** 20S proteasome activity of NSC34 cell lysates transfected with indicated plasmids were measured with 20S proteasome specific substrate fluorogenic peptides Suc-LLVY-AMC. Time-dependent changes in 20S proteasome activity were presented as line graphs. Data were presented as the mean ± SEM from three independent cell-culture experiments (N = 3 biological replicates). Statistical significance was analyzed using two-way ANOVA to compare groups across multiple time points

To further investigate whether the TDP-43-TNKS interaction contributes to the disruption of TNKS/PI31 signaling axis and proteasome dysfunction, we generated a TDP-43 mutant (TDP-43-TBD) harboring H166A and I168A substitutions within the tankyrase-binding motif (RxxΦDG) in the RRM1 domain to disrupt its interaction with TNKS [63]. In the RIPA/urea fractionation assay, compared with TDP-43-FL, TDP-43-TBD predominantly accumulated in the insoluble urea fraction (Fig. 6A) and formed pronounced cytoplasmic aggregates (Fig. 6B). However, TNKS levels in the urea-insoluble fraction were lower in TDP-43-TBD–transfected cells than that in TDP-43-FL–transfected cells (Fig. 6A). These results indicate that disrupting the interaction between TNKS and TDP-43 leads to a significant increase in the aggregation propensity of TDP-43-TBD, yet fails to effectively sequester TNKS into the insoluble fraction. Furthermore, compared with TDP-43-FL, we found a markedly reduced interaction between TNKS and TDP-43-TBD, accompanied by increased TNKS–PI31 binding in co-immunoprecipitation analysis, suggesting that TDP-43-TBD has a reduced ability to sequester TNKS and thereby permits greater TNKS availability for interaction with PI31 (Fig. 6C). We further assessed the impact of TDP-43-TBD on 20S proteasome activity, even though TDP-43-TBD is more prone to form insoluble aggregates than TDP-43-A315T, TDP-43-TBD did not impair 20S proteasome activity as significant as TDP-43-A315T (Fig. 6D). These findings indicate that aggregation of TDP-43 alone is insufficient to disrupt proteasome function. Instead, the interaction between TDP-43 and TNKS is an important contributor to TNKS sequestration, the impairment of the TNKS/PI31 signaling axis, and the subsequent inactivation of the proteasome.

### TNKS enzymatic activity is critical for preserving the TNKS–PI31–20S proteasome regulatory axis in TDP-43 neuropathy

**Fig. 7:**
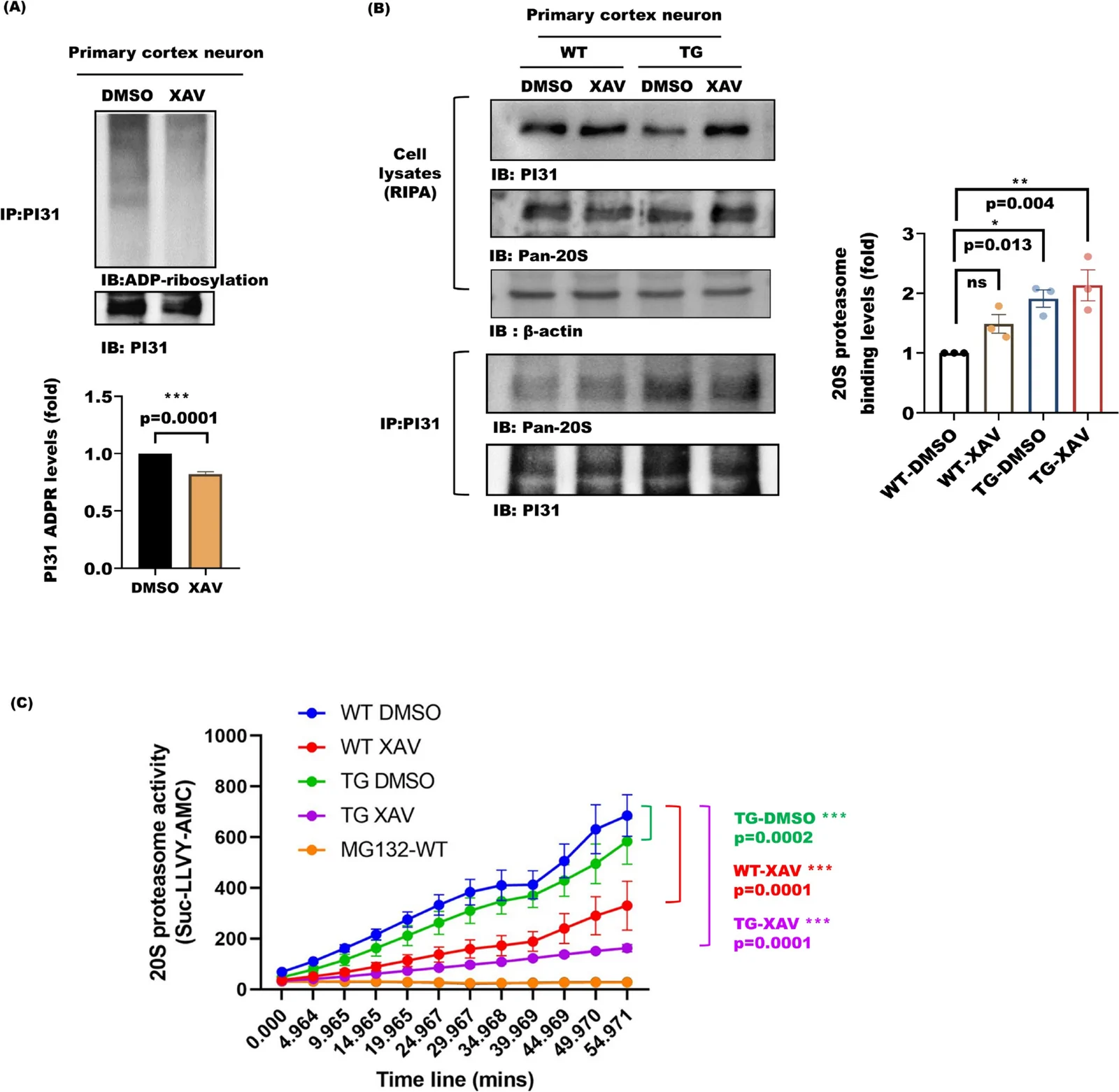
TNKS enzymatic activity is critical for preserving the TNKS–PI31–20S proteasome regulatory axis in TDP-43 neuropathy. **A** Primary cortex neurons derived from non-Tg wild-type mice treated with DMSO or 10 µM XAV939 for 15 h were immunoprecipitated with PI31 antibody and immunoblotted with anti-ADP-ribose antibodies. Western blotting and bar graphs showed lower PI31 ADP-ribosylation levels of PI31 ADP-ribosylation in XAV939-treated groups compared with DMSO control groups. Pull down PI31 ADP-ribosylation levels were normalized to PI31. Data were presented as the mean ± SEM from three independent cell-culture experiments (N = 3–4 biological replicates). Comparisons between two groups were performed using a unpaired two-tailed Student's t-test. **B** Primary cortex neurons derived from non-Tg wild-type and Prp-TDP-43^A315T^ mice treated with DMSO or 10 µM XAV939 were immunoprecipitated with PI31 antibody and immunoblotted with anti-Pan 20S and PI31 antibodies. Western blotting and bar graphs showed the interaction levels between PI31 and 20S proteasome in DMSO or XAV939-treated groups. Pull down 20S proteasome levels were normalized to PI31. Data were presented as the mean ± SEM from three independent cell-culture experiments (N = 3 biological replicates). Groups were compared using one-way ANOVA.** C** 20S proteasome activity of primary cortex neurons derived from non-Tg wild-type and Prp-TDP-43^A315T^ mice treated with DMSO or 10 µM XAV939 for 15 h were measured with 20S proteasome specific substrate fluorogenic peptides Suc-LLVY-AMC. Time-dependent changes in 20S proteasome activity were presented as line graphs Data were presented as the mean ± SEM from three independent cell-culture experiments (N = 3 biological replicates). Statistical significance was analyzed using two-way ANOVA to compare groups across multiple time points

Our findings suggest that pathological TDP-43 disrupts proteasome activity by interfering with TNKS-mediated PI31 ADP-ribosylation, supporting a role for TNKS enzymatic activity in maintaining TNKS/PI31/20S proteasome function in TDP-43 neuropathies. To further confirm this point, we pharmacologically inhibited TNKS enzymatic activity using the selective TNKS inhibitor XAV939, which is a cell-permeable, selective TNKS inhibitor that blocks TNKS-mediated PI31 ADP-ribosylation [22, 64]. Primary cortical neurons derived from wild-type and Prp-TDP-43^A315T^ mice brain were treated with XAV939, and PI31 ADP-ribosylation, PI31-20S proteasome interaction, and proteasome activation were analyzed. Consistent with previous findings [22], wild-type primary cortical neurons treated with XAV939 showed significantly reduced ADP-ribosylation of PI31 compared with the DMSO controls (Fig. 7A). Increased PI31 binding to the 20S proteasome (Fig. 7B) and reduced 26S proteasome activity (Fig. 7C) in both wild-type and Prp-TDP-43^A315T^ mice primary cortical neurons treated with XAV939 were observed as compared to those without XAV939 treatment, which recapitulates the effects of TDP-43-A315T mutant Prp-TDP-43^A315T^ mice observed in 7B and 7C. In addition, increased insoluble TDP-43 oligomers and polymers in the urea fraction were also observed in N2a or 293 T cells transfected with pathological GFP-TDP-43-A315T or GFP-TDP-25 and treated with XAV939, compared with DMSO-treated controls (Fig. S4 and S5 (Additional file 1)). Taken together, these results suggest that inhibition of TNKS- mediated PI31 ADP-ribosylation enzymatic activity by XAV939 in wild-type and Prp-TDP-43^A315T^ cortical neurons replicates the effect of pathological TDP-43-mediated disruption of the TNKS/PI31/proteasome regulatory axis, leading to more toxic oligomeric TDP-43 formation in TDP-43 neuropathies.

### TNKS inhibition recapitulates pathological TDP-43-mediated impairment of axonal 20S proteasome transport

**Fig. 8:**
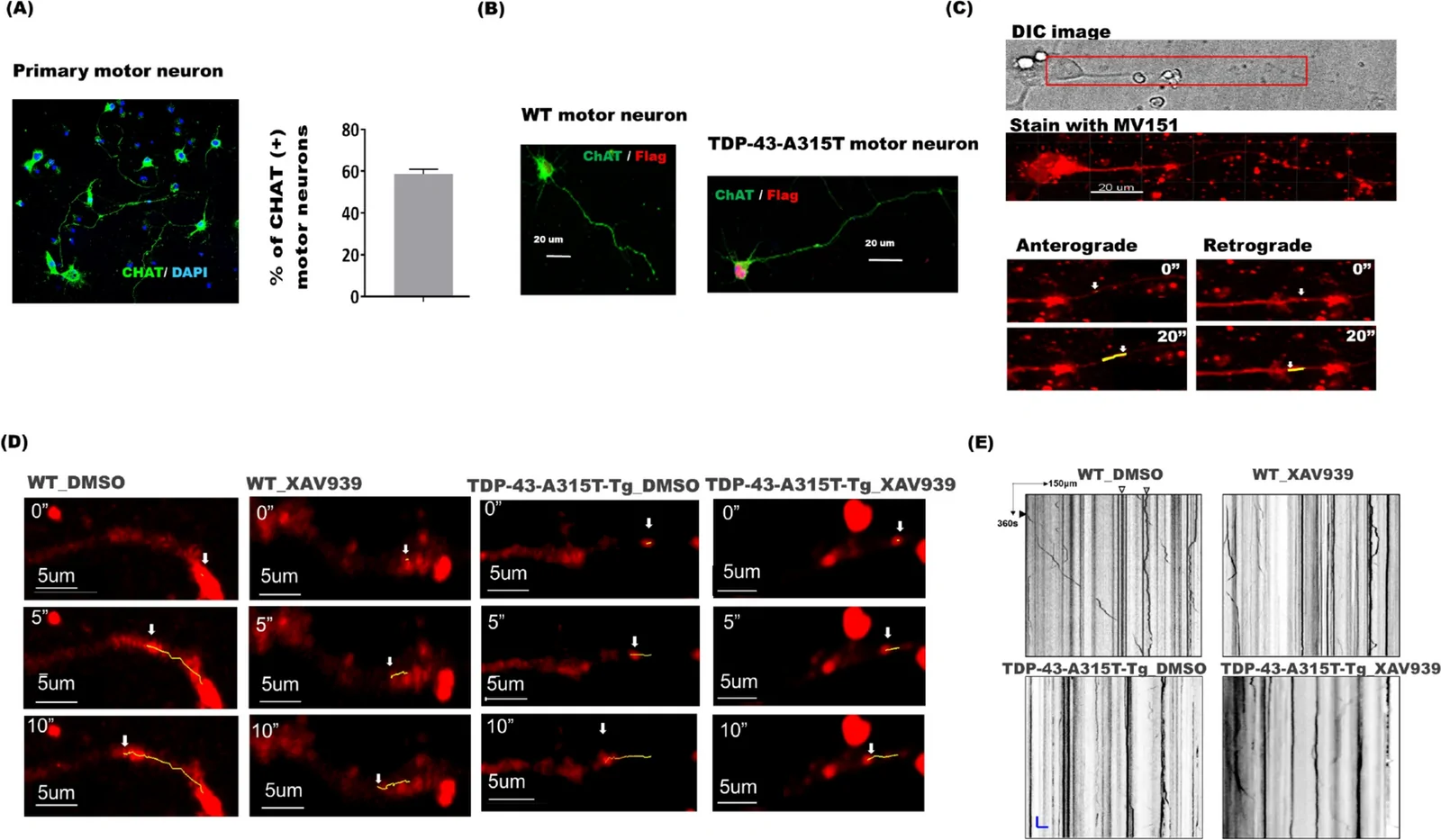
The role of TNKS in 20S proteasome movement within axon under normal condition and in TDP-43 neuropathies. **A**, **B** Primary motor neurons were extracted from E12.5 embryos of non-transgenic (Non-Tg) wild-type littermates and Prp-TDP-43^A315T^ mice spinal cord and characterized by immunofluorescence staining at DIV7 using the specific motor neuron markers CHAT in green. Around 60–70% of cells were motor neurons. Data were obtained from three independent biological replicates (N = 3 mice) with 50 technical measurements per condition (n = 50 cells). Statistical analyses were performed using pooled technical measurements. **C** Live-cell imaging of primary motor neurons extracted from E12.5 embryos of non-transgenic (Non-Tg) wild-type littermates and Prp-TDP-43^A315T^ mice spinal cord was performed at DIV6 to examine the axonal proteasome movement. The top shows the corresponding DIC images of neurons from which specific axonal regions (red boxes) were selected for real-time analysis. Representative real-time images of endogenous 20S proteasome (arrows with yellow tracking lines) moving along a selected neuronal axon of a DIV6 primary motor neuron in the anterograde (away from soma) and retrograde (toward soma) directions along the axon within 20 secs. Scale bars, 20 μm. **D** Representative images showing the time-dependent movement of 20S proteasomes along axons within a 10 s period. Arrowheads and yellow tracking lines indicate the direction and trajectory of proteasome movement. Scale bars, 5 μm. **E** Kymographs tracing axonal proteasomes in live primary motor neurons derived from non-Tg wild-type and Prp-TDP-43^A315T^ mice treated with or without 2 µM XAV939 (see Materials and Methods). X-axis indicates the axon length of 150 µm; Y-axis represents a time period of 360 secs. The stationary (white arrowhead), oscillatory (gray arrowhead), and motile (black arrowhead) 20S proteaomse were shown here. Data were obtained from three independent biological replicates (N = 3 mice) with 8–32 technical measurements per condition (n = 8–32 cells). Statistical analyses were performed using pooled technical measurements

**Fig. 9:**
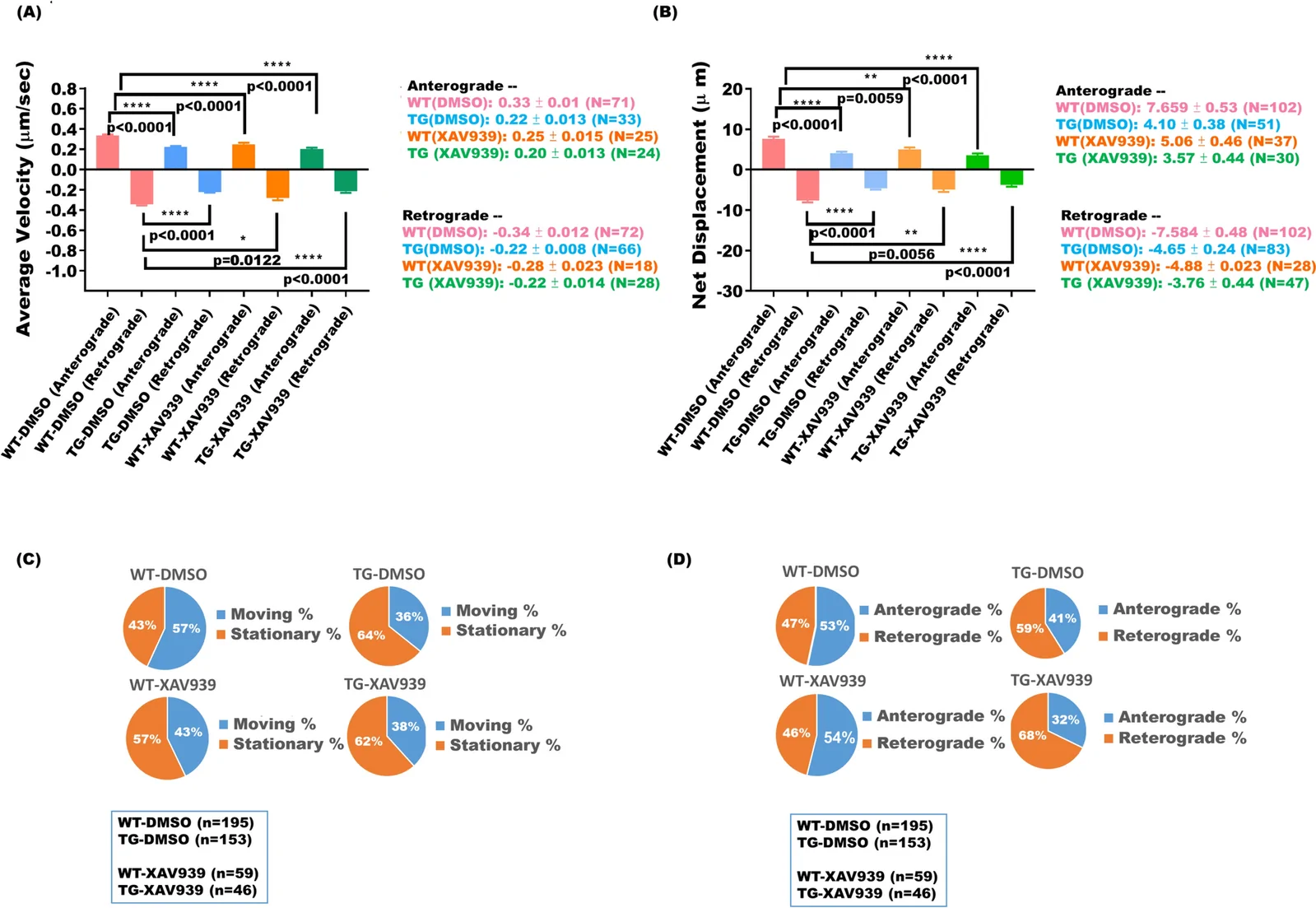
TNKS inhibition attenuates axonal 20S proteasome transport and recapitulates TDP-43 neuropathy-associated defects. **A**, **B** Comparison of average velocities (**A**) and net displacements (**B**) of axonal 20S proteasome in live primary motor neuron derived from non-Tg wild-type and Prp-TDP-43^A315T^ mice treated with or without 2 µM XAV939 (see Materials and Methods). Bar diagram represented the average anterograde and retrograde displacements (μm) and velocities (μm/s) of proteasomes. Data were presented as the mean ± SEM from at least three independent biological replicates (N = 3–4 mice) with 18–102 technical measurements per condition (n = 18–102 individual 20S proteasome granules). Statistical analyses were performed using pooled technical measurements. Groups were compared using Ordinary one-way ANOVA. **C** 20S proteasomes with velocity higher than 0.1 µm/s within a 10–20 s tracking time-period (0.5 s/frame) were defined as moving in a single direction, while those moving slower than 0.1 µm/s were considered as stationary. The pie charts showed the proportions (%) of stationary versus uni- or bi-directionally moving 20S proteasomes. Data were obtained from at least three independent biological replicates (N = 3 − 4 mice) with 46 − 195 technical measurements per condition (n = 46 − 195 individual 20S proteasome granules). Statistical analyses were performed using pooled technical measurements. The proportions of stationary and moving proteasomes, as well as the proportions of anterograde and retrograde movements, were compared using Fisher's exact test (*p = 0.0045 between WT-DMSO and TG-DMSO; **p = 0.01 between WT-DMSO and TG-XAV939). **D** The pie charts showed the proportions (%) of and anterograde versus retrograde moving 20S proteasomes. Data were obtained from at least three independent biological replicates (N = 3 − 4 mice) with 46 − 195 technical measurements per condition (n = 46 − 195 individual 20S proteasome granules). Statistical analyses were performed using pooled technical measurements. The proportions of stationary and moving proteasomes, as well as the proportions of anterograde and retrograde movements, were compared using Fisher's exact test (*p = 0.01 between WT-DMSO and TG-DMSO; **p = 0.004 between WT-DMSO and TG-XAV939)

Given that TNKS–mediated PI31 ADP-ribosylation activity is critical for 20S proteasome activity regulation in TDP-43 neuropathy, we next investigated whether the TNKS/PI31 axis also contributes to the disruption of 20S proteasome transport within axons in the presence of pathological TDP-43. We conducted time-lapse imaging for live-cell fluorescence to study the effect of TNKS on the dynamics of anterograde or retrograde movement of endogenous MV151-labeled 20S proteasome transport in the DMSO or XAV939-treated E12.5 primary motor neuron derived from the spinal cord of non-Tg wild-type control or Prp-TDP-43^A315T^ Tg mice. Here we use CHAT as a motor neuron marker and found that around 60% of cells were CHAT (+) motor neurons in both non-Tg wild-type control or Prp-TDP-43^A315T^ Tg mice (Fig. 8A, B). Endogenous 20S proteasome labeled with MV151 were distributed throughout the soma and axon and showed anterograde and retrograde movement within axons (Fig. 8C). Live-cell fluorescent imaging of motor neurons from non-Tg wild-type control or Prp-TDP-43^A315T^ Tg mice treated with or without XAV939 exhibited different retrograde transport patterns of 20S proteasome within axon (Fig. 8D). Kymograph shows stationary (white arrowhead), oscillatory (gray arrowhead), and motile (black arrowhead) 20S proteasome (150 μm and 360 s) (Fig. 8E). Consistent to our results in primary cortex neurons transfected with GFP-TDP-25 or GFP-TDP-43-FL (Fig. 3 and S3 (Additional file 1)), the proteasomes in Prp-TDP-43^A315T^ Tg mice were less dynamic than those in non-Tg wild-type control motor neurons (Figs. 8D, E and 9). In Prp-TDP-43^A315T^ Tg mice motor neurons, the average velocity and net displacement showed a significant decreased trend in both retrograde and anterograde direction, as compared with those in wild-type motor neurons, further confirming that pathological TDP-43 interrupts 20S proteasome transport in axon (Fig. 9A, B) (Movies 1 to 4 for non-Tg wild-type mice treated with DMSO; and Movies 5 to 8 for Prp-TDP-43^A315T^ Tg mice treated with DMSO). As similar as that in Prp-TDP-43^A315T^ Tg mice, when TNKS activity was inhibited by XAV939 in non-Tg wild-type control or Prp-TDP-43^A315T^ Tg mice motor neurons, diminished average velocity and net displacement were also significant, as compared to those with DMSO treatment (Fig. 9A, B) (Movies 9 to 12 for non-Tg wild-type mice treated with XAV939; and Movies 13 to 16 for Prp-TDP-43^A315T^ Tg mice treated with XAV939). In addition, in Prp-TDP-43^A315T^ Tg mice, the % of moving 20S proteasome was dramatically reduced in Prp-TDP-43^A315T^ Tg mice primary motor neuron (36%) as compared to non-Tg wild-type control (57%) (Fig. 9C). Similar to the effect in Prp-TDP-43^A315T^ Tg mice, in both non-Tg wild-type control and Prp-TDP-43^A315T^ Tg mice primary motor neuron treated with XAV939, the % of moving 20S proteasome was also reduced (43% in non-Tg wild-type control mice and 38% in Prp-TDP-43^A315T^ Tg mice) as compared to DMSO-treated non-Tg wild-type control ones (57%) (Fig. 9C). Interestingly, we observed an increased proportion in retrograde movement of 20S proteasome in Prp-TDP-43^A315T^ Tg mice (59%), as compared to those in non-Tg wild-type control (47%) (Fig. 9D). Also, comparing to DMSO-treated wild-type ones (47%), increased proportion of retrograde movement of 20S proteasome (68%) was observed only in XAV939-treated primary motor neuron of Prp-TDP-43^A315T^ Tg mice, but not in XAV939-treated wild-type mice (46%) (Fig. 9D). Taken together, our results demonstrate that inhibition of TNKS activity by XAV939 recapitulates the pathological TDP-43-mediated reduction in axonal 20S proteasome motility in Prp-TDP-43^A315T^ Tg mice, suggesting that pathological TDP-43 disrupts the TNKS/PI31-dependent proteasome transport cascade and thereby exacerbates proteasome dysfunction and impaired protein clearance.

### TNKS inhibition recapitulates pathological TDP-43-induced accumulation of TDP-43, PI31, and proteasomes in the proximal axons of motor neurons

**Fig. 10:**
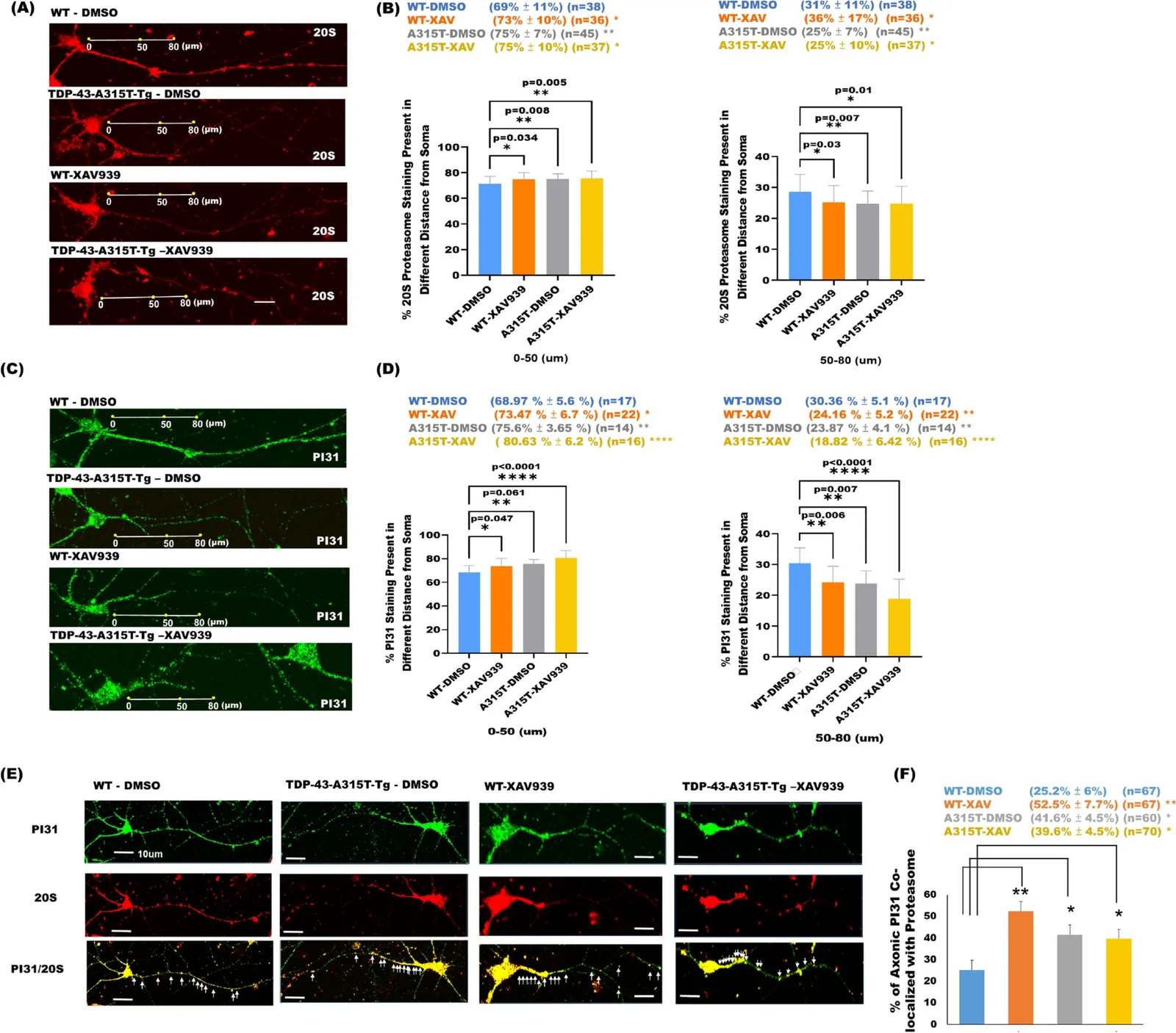
TNKS inhibition recapitulates pathological TDP-43-induced accumulation of TDP-43, PI31, and proteasomes in the proximal axons of motor neurons. **A**–**D** Primary motor neurons were extracted from E12.5 embryos of non-Tg wild-type (WT) and Prp-TDP-43^A315T^ Tg mice and treated with DMSO or 2 µM XAV939 for 15 h. Representative fluorescence images show the fluorescence staining of MV151-labeled 20S proteasomes (red) (**A**) and PI31 (green) (**C**). Axonal regions located 0–50 μm (proximal) and 50–80 μm (distal) from the soma were analyzed. **B**, **D** The bar graphs represented the percentage of fluorescence intensity of MV151-labeled 20S proteasomes (**B**) and PI31 (**D**) within the proximal (0–50 μm) and distal (50–80 μm) axonal regions, relative to the total fluorescence intensity of the 0–80 μm axonal region. Data were presented as the mean ± SEM from at least three independent biological replicates (N = 3–4 mice), with 37–45 technical measurements per condition analyzed for 20S proteasome measurements (n = 37–45 individual axon measurements) and 14–22 technical measurements per condition analyzed for PI31 measurements (n = 14–22 individual axon measurements). Statistical analyses were performed using pooled technical measurements. Groups were compared using Ordinary one-way ANOVA. (**E**) Fluorescence images of primary motor neurons derived from non-Tg wild-type (WT) and Prp-TDP-43^A315T^ Tg mice, treated with DMSO or XAV939, showed colocalization patterns between PI31 (green) and MV151-labeled 20S proteasomes (red). The colocalization of PI31 and 20S proteasome was indicated in yellow. **(F)** The bar graphs represented the percentage of % of PI31 (green) co-localization with 20S proteasome (red) within axon. Data were presented as the mean ± SEM from at least three independent biological replicates (N ≥ 3 mice), with 60–70 technical measurements per condition (n = 60–70 individual axon measurements). Statistical analyses were performed using pooled technical measurements. Groups were compared using Ordinary one-way ANOVA

To determine whether 20S proteasome dysfunction contributes to altered TDP-43 subcellular localization within neurons, we analyzed its spatial distribution in primary motor neurons. Similar to previous findings, in the motor neurons of Prp-TDP-43^A315T^ Tg mice, TDP-43 mainly accumulates in the cell body and proximal axons, while its content is reduced or almost absent in the distal axons (Fig. S6) [13]. In addition to TDP-43, we observed that MV151-labeled 20S proteasomes were predominantly clustered in the proximal axons (0-50 μm from the cell body), while rarely appearing in the distal axons (50-80 μm from the cell body) in Prp-TDP-43^A315T^ Tg mice (Fig. 10A, B). Consistently, PI31 also exhibited proximal axonal enrichment (Fig. 10C, D), and the proportion of PI31 co-localized with MV151-labeled 20S proteasome in axon was significantly increased in Prp-TDP-43^A315T^ Tg mice motor axon as compared to non-Tg wild-type control mice (Fig. 10E, F). Moreover, upon TNKS inhibition by XAV939, not only TDP-43 but also MV151-labeled 20S proteasomes and PI31 exhibited pronounced accumulation in the soma and proximal axons in both XAV939-treated non-Tg wild-type control and Prp-TDP-43^A315T^ Tg motor neurons as compared to those with DMSO treatment (Fig. S6 (Additional file 1) and 10A–D), and the % of axonic PI31 co-localizaed with MV151-labeled 20S proteasome was also significantly increased in XAV939-treated non-Tg wild-type control mice and Prp-TDP-43^A315T^ Tg mice motor neuron as compared with DMSO treated controls, resembling the distribution observed in Prp-TDP-43^A315T^ Tg mice overexpressing the pathological TDP-43-A315T mutants (Fig. 10E, F). Collectively, these findings demonstrate that pathological TDP-43 disrupts the 20S proteasomes transport in Prp-TDP-43^A315T^ Tg mice, leading to the accumulation of 20S proteasomes, PI31, and TDP-43 in the soma and proximal axons of motor neurons in TDP-43 neuropathies. While inhibition of TNKS activity by XAV939 recapitulates the defects in proteasome transport and disruption of protein homeostasis caused by pathological TDP-43. Although the increased relative proportion of retrograde transport events may represent a compensatory response to these proteins accumulation in proximal axons, impaired proteasome activity ultimately contributes to protein accumulation and disruption of axonal proteostasis.

## Discussion

**Fig. 11:**
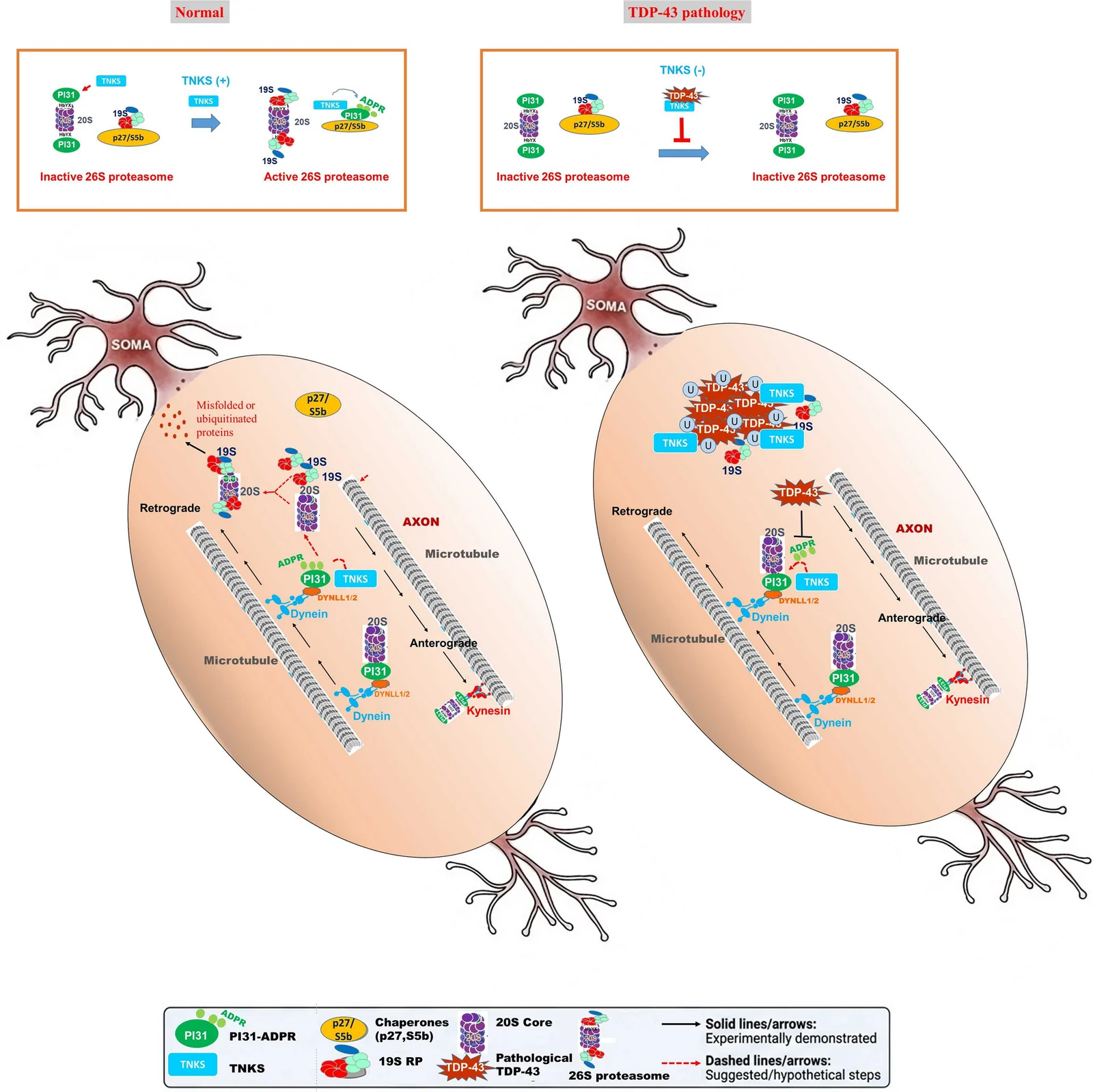
The TNKS-PI31 signal cascade involved in proteasome activity and axonal transport of 20S proteasome under normal condition and TDP-43-related neuropathies. Under physiological conditions, TNKS-mediated ADP-ribosylation of PI31 reduces the affinity of PI31 for the 20S core particle while promoting its interaction with chaperone proteins such as p27 and S5b. This facilitates the release of the 20S proteasome from inhibitory PI31 binding and promote assembly of the 20S core with the 19S regulatory particle to form an active 26S proteasome. During axonal transport, PI31 functions as an adaptor linking the 20S proteasome to the dynein motor complex, thereby facilitating microtubule-based trafficking. Upon arrival at appropriate subcellular compartments (e.g., soma, axon, or synapse), TNKS-mediated ADP-ribosylation of PI31 may facilitate the release of the 20S proteasome from PI31 and thereby permit local assembly of the 26S proteasome. Efficient proteasome assembly and distribution would be expected to support the degradation of misfolded and ubiquitinated proteins and maintenance of neuronal proteostasis. Under physiological conditions, balanced anterograde and retrograde transport may contribute to appropriate proteasome distribution and turnover across neuronal compartments. In TDP-43-related neuropathology, excessive or misfolded TDP-43 aberrantly associates with TNKS and sequesters TNKS into insoluble aggregates. This reduces the availability of functional TNKS for interaction with PI31, resulting in decreased TNKS-mediated ADP-ribosylation of PI31. Consequently, PI31 remains more tightly associated with the 20S proteasome, while the 19S regulatory particle may remain associated with chaperone proteins such as p27 and S5b, thereby impairing 19S/20S assembly. Defective TNKS-mediated PI31 regulation may contribute to impaired proteasome trafficking and local proteasome assembly. Although PI31 can mediate the transport of 20S proteasomes along microtubules, TNKS-mediated PI31 ADP-ribosylation may facilitate the release of transported 20S proteasomes at target locations. Impaired TNKS-mediated PI31 ADP-ribosylation under TDP-43 pathology may therefore reduce proteasome unloading and, consequently, limit subsequent local assembly of active 26S proteasomes. These alterations may reduce proteolytic capacity and promote the accumulation of misfolded and ubiquitinated proteins, ultimately contributing to proteotoxic stress and neuronal dysfunction

Our findings highlight the detrimental effects of pathological TDP-43 on proteasome activity and axonal transport, both of which are central to neurodegenerative diseases like ALS and FTLD. Here we demonstrate that overexpression of pathological oligomeric TDP-43, either wild-type, A315T mutant or truncated forms of TDP-43, leads to a significant reduction in proteasome activity, which in turn leads to accumulation of polyubiquitylated proteins. Additionally, we provide a comprehensive picture of how pathological TDP-43 affects the axonal transport of proteasomes through interfering TNKS/PI31 signal axis. Our live-cell imaging of primary cortex or motor neurons revealed that overexpression of TDP-43 disrupts the movement of 20S proteasome within axons, reducing their velocity and net displacement, suggesting that TDP-43 impairs the transport of proteasomes to appropriate subcellular compartments which is essential for maintaining local protein homeostasis and critical for the removal of damaged proteins. The inhibition of proteasome transport leads to a failure to clear toxic protein aggregates in axons, a characteristic feature of ALS, and ultimately contributes to neuronal dysfunction (Fig. 11).

Oligomerization of TDP-43 via its RRMs or C-terminal amyloid core is promoted by pathological mutations, post-translational modifications, reduced RNA-binding capacity, dysregulated LLPS, or oxidative stress [65]. Early-stage TDP-43 oligomers have been identified as a dynamic intermediate species that drive the transition toward aggregated and inclusion-prone forms of TDP-43 [43], leading to diverse cytotoxic effects such as mitochondrial dysfunction, impaired protein homeostasis, neuroinflammation, and synaptic disruption [58, 59]. Although TDP-43-FL and TDP-43A315T did not form visible cytoplasmic aggregates in our cell culture or Prp-TDP-43A315T transgenic mouse models (Fig. 1 and [50]), accumulating evidence suggests that, in addition to filamentous aggregates, soluble TDP-43 oligomers represent major toxic species in TDP-43 proteinopathies. We found that the oligomeric forms of TDP-43 are sufficient to impair proteasome activation and trafficking, highlighting a critical role for oligomeric forms of TDP-43 in disrupting proteasome transport in motor neurons. This underscores the importance of targeting this pathological TDP-43 oligomeric species in therapeutic strategies for TDP-43 proteinopathies such as ALS and FTLD.

Here, we observed an increased relative proportion of retrograde proteasome transport in the presence of pathological TDP-43 in Prp-TDP-43A315T Tg mice. In these mice, phosphorylated TDP-43 oligomers disrupt PI31-mediated 20S proteasome transport in axons, accompanied by the accumulation of PI31, 20S proteasomes, and pathological TDP-43 in the soma and proximal axons of motor neurons. The increased relative proportion of retrograde proteasome movements may reflect a compensatory response to proteotoxic stress, potentially facilitating the redistribution of proteasomes toward the soma and proximal axons. However, because absolute retrograde flux and proteasome delivery to the soma were not directly measured, this interpretation remains tentative. and alternative explanations, such as impaired anterograde transport or altered motor-dependent trafficking, cannot be excluded. A similar shift in the relative proportion of retrograde transport was observed following TNKS inhibition by XAV939, suggesting that disruption of the TNKS–PI31 axis may contribute to the altered proteasome trafficking associated with pathological TDP-43. Despite this potential adaptive response, proteasome function remains compromised, which may limit the capacity of proteasomes to efficiently degrade accumulated substrates and restore proteostasis. Consequently, PI31, 20S proteasomes, and TDP-43 aggregates accumulate in the soma and proximal axons. Mechanistically, our findings suggest that pathological TDP-43 disrupts the TNKS/PI31/20S proteasome regulatory axis, thereby altering axonal proteasome trafficking and impairing proteasome activation. Together, these findings suggest that disruption of the TNKS–PI31–proteasome axis contributes to impaired axonal proteostasis in TDP-43-associated pathology.

TNKS1 and TNKS2 are two homologous proteins that belong to the PARPs family, recently named ADP-ribosyltransferase (ARTD), which control a wide range of cellular processes, including Wnt-β-catenin signaling pathway, telomere length maintenance, DNA damage repair and glucose homeostasis and vesicle trafficking [66–68]. Previous research by McGurk et al. reported that TNKS1/2 promotes TDP-43 accumulation in the cytoplasm by interacting with TDP-43 and inhibiting TDP-43 ubiquitination and degradation via the proteasome in the nucleus [63, 69]. While this seems to contradict our finding that TNKS positively regulates proteasome function through the TNKS-PI31 axis, our experimental results actually provide a more precise explanation for McGurk et al.'s findings. We found that under conditions of TDP-43 overexpression, pathological TDP-43 sequesters TNKS into cytosolic insoluble complexes. Thus, rather than TNKS acting as a primary driver that traps TDP-43, our results suggest that pathological TDP-43 acts upstream to trap and functionally inactivate TNKS. This sequestration impairs TNKS-mediated ADP-ribosylation of PI31, leading to persistent PI31 binding to the 20S proteasome, defective 19S–20S assembly, and reduced 26S proteasome activity. In addition to PI31, TDP-43 has also been demonstrated as the binding target of TNKS through its canonical TNKS-binding motif (RxxΦDG) in the RRM1 domain on TDP-43 [63, 69]. As we know that TNKS mediates ADP-ribosylation of PI31, but whether TDP-43 is also a substrate of TNKS and can be ADP-ribosylated by TNKS have never been addressed, even though PAR (poly-ADP-ribosylation)-binding motif (PBM) has been identified on TDP-43 protein [69]. McGurk’S group found that mutation of the PBM on TDP-43 reduced both of its polyubiquitination and cytosolic accumulation, implying that PARylation of TDP-43 might facilitate ubiquitination-dependent proteasome degradation of mutant TDP-43. They also found that PARylation of TDP-43 facilitates its recruitment into stress granules, where it is protected from pathological phosphorylation and aggregation during short-term stress, while prolonged stress causes TDP-43 to exit the stress granules and accumulate in cytosol to form toxic and insoluble aggregates [63, 69]. However, whether TDP-43 is PARylated by TNKS was not addressed in their study, and the impacts of TNKS-mediated TDP-43 PARylation on proteasome degradation, nuclear translocation and stress granules formation of TDP-43 were not thoroughly explored yet. Several questions remain elucidative and exploring the comprehensive underlying detailed mechanisms of TNKS-mediated ADP-ribosylation of TDP-43 under normal conditions and TDP-43 neuropathy would provide a specific way for targeting the pathological hallmark TDP-43 to proteasome degradation via TNKS-PI31 signal cascade, rather than indiscriminately activating the proteasome and causing unnecessary side effect.

While our findings establish a functional link between pathological TDP-43, the TNKS/PI31/proteasome axis, and proteostasis dysfunction, several important questions remain to be addressed. Although the complementary use of cellular models and Prp-TDP-43^A315T^ transgenic mice enabled us to examine the TNKS/PI31/proteasome axis across multiple experimental systems, these models cannot fully recapitulate the genetic, cellular, and pathological heterogeneity of human ALS and FTLD. Thus studies using patient-derived neuronal systems may further clarify how distinct pathological TDP-43 species contribute to proteasome dysfunction and proteostasis failure in human ALS and FTLD diseases. Moreover, our findings suggest that the TDP-43–TNKS interaction may represent a therapeutically relevant intervention point. Future studies using genetic approaches and patient-derived iPSC motor neurons will help further validate the TNKS/PI31/proteasome axis across disease-relevant systems. In parallel, the design of peptides that selectively modulate the TDP-43–TNKS interaction may provide a valuable strategy to determine whether restoration of TNKS/PI31 signaling can enhance pathological TDP-43 clearance, preserve neuronal function, and improve proteostasis while maintaining appropriate proteasome regulation. Further characterization of the efficacy, selectivity, pharmacological properties, and long-term effects of such approaches will be essential for advancing the translational potential of targeting this pathway. Collectively, our findings identify the TNKS/PI31/proteasome axis as a mechanistically relevant pathway linking pathological TDP-43 to proteasome dysfunction and impaired axonal proteostasis, providing a rationale for further investigation of this pathway as a potential therapeutic target in TDP-43-associated neurodegenerative diseases.

## Conclusions

We identify a functional TNKS/PI31/20S proteasome axis that contributes to the regulation of proteasome activity and axonal trafficking, with important implications for TDP-43 clearance and neuronal proteostasis. Under physiological conditions, TNKS-mediated PI31 ADP-ribosylation supports proteasome assembly and contributes to the regulation of bidirectional proteasome transport along axons. Under pathological conditions, excessive TDP-43 sequesters TNKS into insoluble complexes, reducing its interaction with PI31 and impairing PI31 ADP-ribosylation, proteasome assembly, and proteasome transport, thereby promoting protein accumulation. Our findings indicate that TNKS enzymatic activity contributes not only to proteasome activation but also to efficient axonal proteasome transport. Pharmacological inhibition of TNKS using XAV939 recapitulates key features observed in TDP-43 models, including those expressing truncated TDP-25 or TDP-43-A315T, resulting in reduced PI31 ADP-ribosylation, impaired 19S–20S proteasome assembly, and decreased 26S proteasome activity. This disruption is accompanied by defects in proteasome transport, including reduced velocity, net displacement, and motility, together with the accumulation of 20S proteasomes and PI31 in the soma and proximal axons. Importantly, our findings suggest that modulating the TNKS/TDP-43 interaction may represent a potential therapeutic strategy to preserve TNKS/PI31 signaling and proteasome function, thereby supporting the clearance of pathological TDP-43. Collectively, these findings identify the TNKS/PI31/proteasome axis as a potential therapeutic target in TDP-43-associated neurodegenerative diseases (Fig. 11).

## Supplementary Information

Additional file1 (PDF 6370 kb)

Moive A - GFP (MP4 523 kb)

Movie B-GFP-TDP-25 (MP4 694 kb)

Movie C-GFP-TDP-FL (MP4 4099 kb)

Movie 1 -WT (DMSO) (MP4 19512 kb)

Movie 2 -WT (DMSO) (MP4 19269 kb)

Movie 3 -WT (DMSO) (MP4 11673 kb)

Movie 4 -WT (DMSO) (MP4 22048 kb)

Movie 5 -Tg (DMSO) (MP4 7134 kb)

Movie 6 -Tg (DMSO) (MP4 6554 kb)

Movie 7 -Tg (DMSO) (MP4 10888 kb)

Movie 8 -Tg (DMSO) (MP4 15420 kb)

Movie 9 -WT (XAV939) (MP4 4787 kb)

Movie 10 -WT (XAV939) (MP4 4477 kb)

Movie 11 -WT (XAV939) (MP4 3059 kb)

Movie 12 -WT (XAV939) (MP4 8652 kb)

Movie 13 - Tg (XAV939) (MP4 2732 kb)

Movie 14 - Tg (XAV939) (MP4 3689 kb)

Movie 15 - Tg (XAV939) (MP4 2970 kb)

Movie 16 - Tg (XAV939) (MP4 4158 kb)

## Data Availability

No datasets were generated or analysed during the current study.

## Abbreviations

ALS: Amyotrophic lateral sclerosis
ARTD: ADP-ribosyltransferase
DYNLL1/2: Dynein light chain LC8-type proteins
FTLD: Frontotemporal lobar degeneration
FTLD-U: Frontotemporal lobar degeneration with ubiquitin-positive inclusions
FRET: Förster Resonance Energy Transfer
FRETc: FRET correction equation
MV151: BODIPY TMR-Ahx3L3VS
LS: Low-salt
PAR: Poly-ADP-ribosylation
PARPs: Poly ADP-ribose polymerase
PBM: PAR (poly-ADP-ribosylation)-binding motif
PI31: Proteasome Inhibitor 31
RRM: RNA‐recognition motifs
SARK: Sarkosyl
SDD-AGE: Semi-denaturing detergent agarose gel electrophoresis
TDP-43: Tar DNA binding protein 43
Tg: Transgenic
TNKS: Tankyrase 1 or 2, also known as PARP5a or 5b, a Poly ADP-ribose Polymerase [PARP] enzyme
TDP-25 and TDP-35: 35KDa and 25 kDa TDP-43 C-terminal fragments
TX: Triton X-100 buffer
UBIs: Ubiquitinated Inclusions
Ub^G76V^^−^GFP: GFP-tagged ubiquitin mutant substrate. The Gly76-to-Val mutation prevents deubiquitination, causing continuous polyubiquitination of GFP
