# Supplementary material for: The TNKS/PI31–proteasome axis as a central node in TDP-43 neuropathies
Source: J Biomed Sci. 2026 Nov 4;33:94. doi: 10.1186/s12929-026-01296-1 (PMC13733214; doi:10.1186/s12929-026-01296-1)
Supplement: Supplementary file 1 [file 12929_2026_1296_MOESM1_ESM.pdf]

Fig. S1

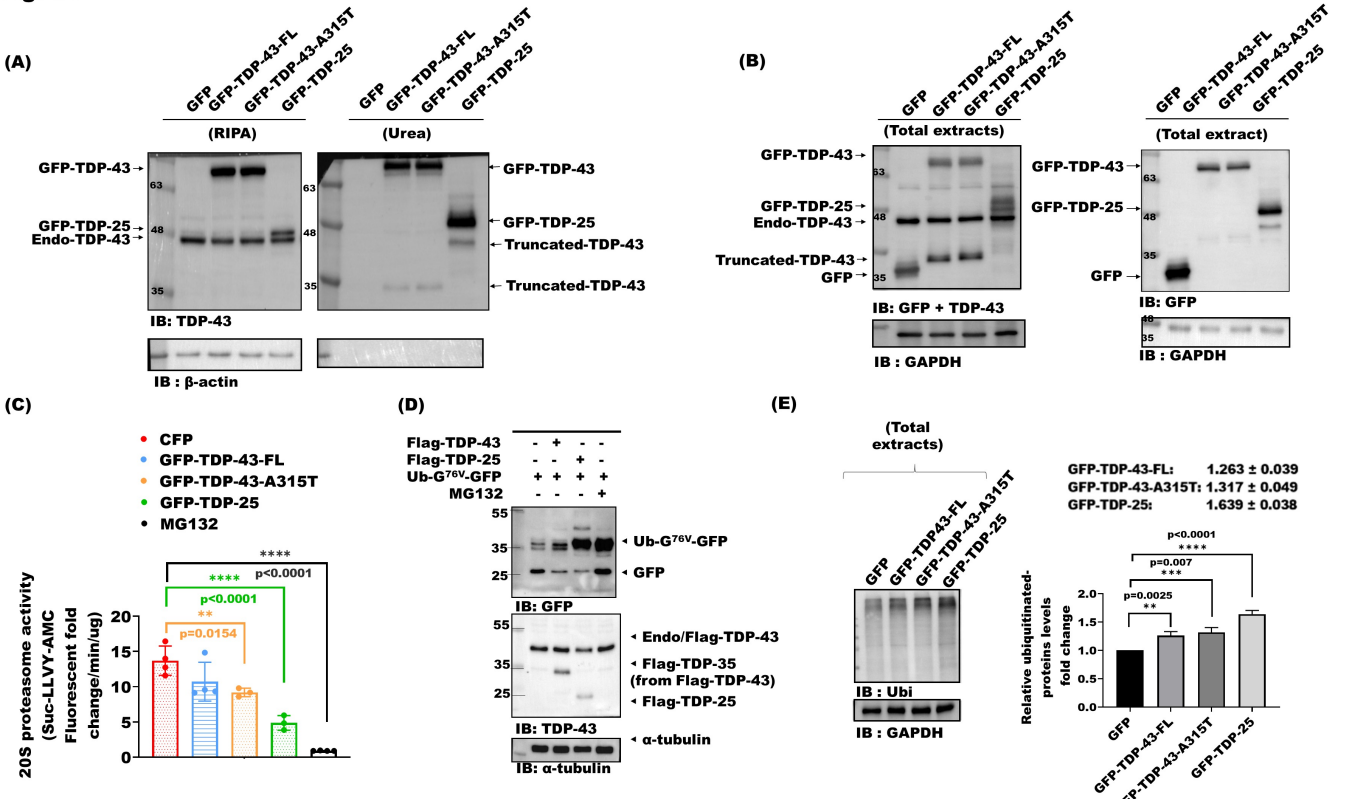

Fig. S1. (A and B) Total amount and relative insolubilities of GFP–TDP-43-FL, TDP-43-A315T mutant and TDP-25 protein species. Equal amount of N2a cells transfected with equal amount of indicated plasmids were separated into total, RIPA- and urea fractions as described in the Materials and Methods. (C) Initial hydrolysis rates of 20S proteasome substrate Suc-LLVY-AMC were calculated from the first 30 minutes of each progress curve based on AMC fluorescence fold change over time (Fig. 1D). Fluorescence at each time point was normalized to the 0-minute value to account for baseline differences across samples. All assays were performed with equal amounts of total protein, so the derived rates reflect relative proteasome activity expressed as fold change per minute under equivalent protein input. These data are presented as individual bar graphs from at least three independent experiments (Groups were compared using Ordinary one-way ANOVA). **(D)** N2a cells transfected with Ub^{G76V}-GFP, Flag-TDP43 and Flag-TDP-25. Cells treated with10 μM MG132 was as negative control. Total cell lysates were immunoblotted with anti-GFP and TDP-43 antibodies. α-tubulin served as a loading control. **(E)** N2a cells transfected with indicated plasmids were then lyzed with urea buffer. Total amount of endogenous ubiquitinated proteins were immunoblotted with anti-Ubi antibodies. GAPDH served as a loading control. Bar diagram represents the relative unbiquitinated proteins levels fold change in cells transfected with GFP or GFP–TDP-43 constructs. Data were presented as the mean ± SEM of at least three independent experiments. (Groups were compared using Ordinary one-way ANOVA)..

Fig. S2.

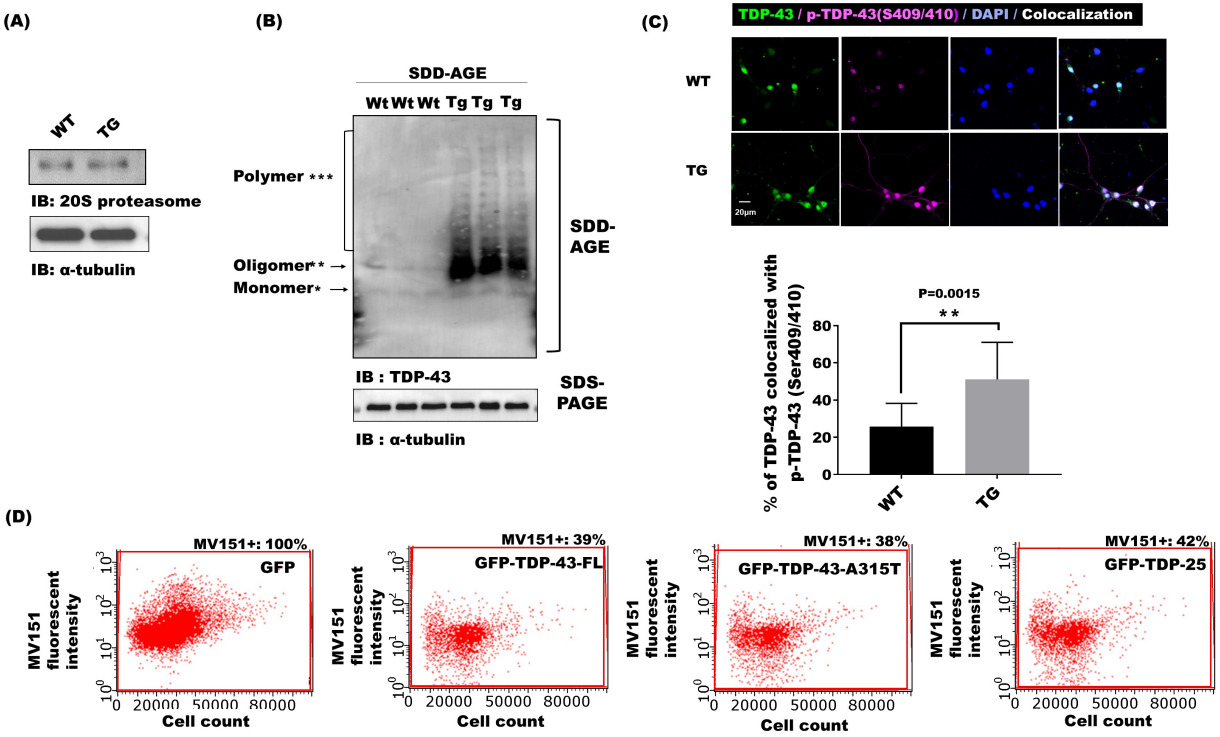

Fig. S2. **(A)** Spinal cord tissues from non-transgenic wild-type littermate controls (WT) and Prp-TDP-43A315T mice (Tg) were lysed, and equal amounts of total protein were subjected to proteasome activity assays (Fig. 2A) and Western blot analysis. Immunoblotting of the 20S proteasome and the loading control α-tubulin confirmed comparable proteasome expression levels and equal protein loading between WT and Tg mice. **(B)** To assess TDP-43 oligomerization and aggregation, spinal cord lysates obtained from non-transgenic (Non-Tg) wild-type littermates and Prp-TDP-43^{A315T} mice were analyzed using SDD-AGE followed by immunoblotting detected with anti-TDP-43 antibody. Distinct bands were observed, corresponding to monomeric TDP-43 (*) and higher molecular weight species, including oligomers (**), polymers or aggregates (***). In parallel, immunoblotting of α-tubulin was used as a loading control to confirm comparable protein input across different transfected conditions. **(C)**. Primary motor neurons derived from E12.5 embryos of non-transgenic (Non-Tg) wild-type littermates and Prp-TDP-43^{A315T} mice were analyzed at DIV7 by immunofluorescence staining using TDP-43 (m/h-TDP-43) antibody (green) and phospho-TDP-43 (Ser409/410) antibody (magenta). Quantification bar graph showing the percentage of colocalization between total TDP-43 and pTDP-43 (Ser409/410) in WT and Prp-TDP-43^{A315T} primary motor neurons. (Independent two groups were compared using unpaired t-test). **(D)** The fluorescence intensity of MV151-labeled 20S proteasomes was further analyzed by flow cytometry. Representative histograms showing the distribution of MV151 fluorescence intensity (X-axis) and cell count (Y-axis) in N2a cells transfected with GFP or GFP–TDP-43 constructs are presented. A total of 5,000 GFP-positive cells were sorted from each sample, and the MV151-positive population was subsequently gated for 20S proteasome activity analysis.

Fig. S3.

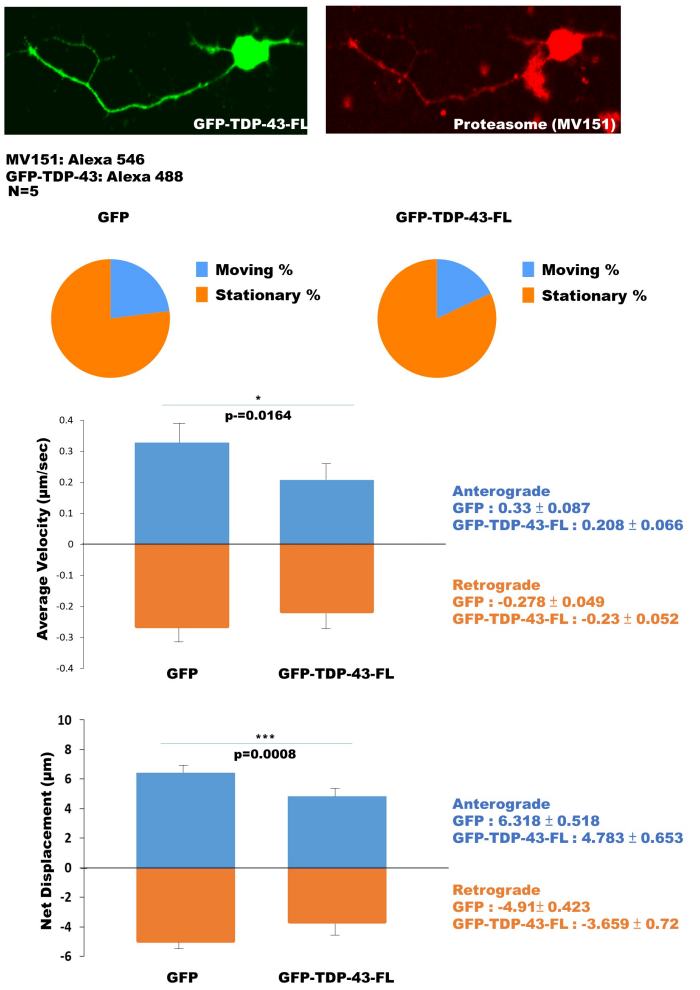

Fig. S3. Overexpression of GFP–TDP-43-FL interrupts the axonal transport of 20S proteasome in wild-type primary cortex neuron. Primary cortical neurons derived from wild-type E16.5 mouse brains were transfected at DIV5 with GFP or GFP–TDP-43-FL for 48 h, and live-cell imaging was performed at DIV7 to examine the effects of GFP–TDP-43-FL on axonal proteasome movement. The pie charts showed the proportions (%) of stationary versus uni- or bi-directionally moving 20S proteasomes.. Data were obtained from three independent biological replicates (N = 5) with 15 technical replicates per condition (n = 15). Statistical significance between groups was assessed using Fisher's exact test. Bar diagram represented the average anterograde and retrograde displacements (μm) and velocities (μm/s) of proteasomes. Data were obtained from three independent biological replicates (N = 5) with 15 technical replicates per condition (n = 15). Comparisons between groups were performed using an unpaired two-tailed Student's t-test.

Fig. S4

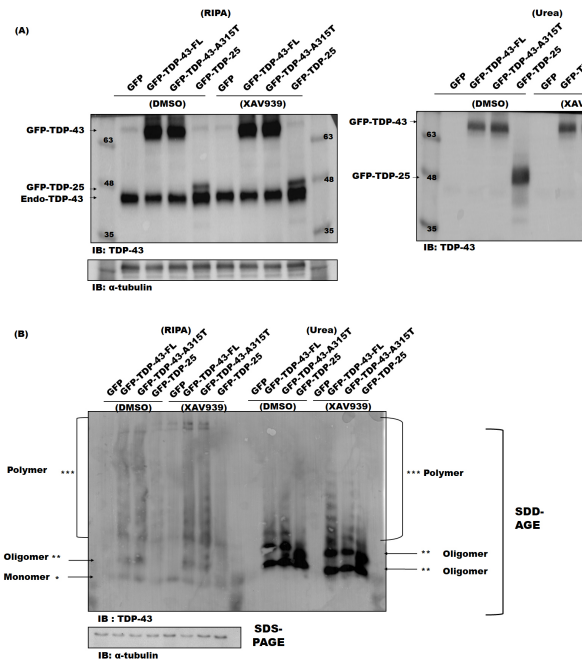

Fig. S4. (A) Relative insolubility and aggregation patterns of GFP–TDP-43-FL, the A315T mutant, and TDP-25 were assessed in the presence or absence of the TNKS-specific inhibitor XAV939. N2a cells transfected with the indicated plasmids were treated with DMSO or 10 μM XAV939 for 15 hours and then fractionated into RIPA-soluble and urea-insoluble fractions. **(B)** SDD-AGE and immunoblotting were used to detect the oligomerization and aggregates patterns of GFP–TDP-43-FL, the A315T mutant, and TDP-25. All blots were probed with TDP-43 antibody (*monomers; **oligomers, ***polymers).

Fig. S5.

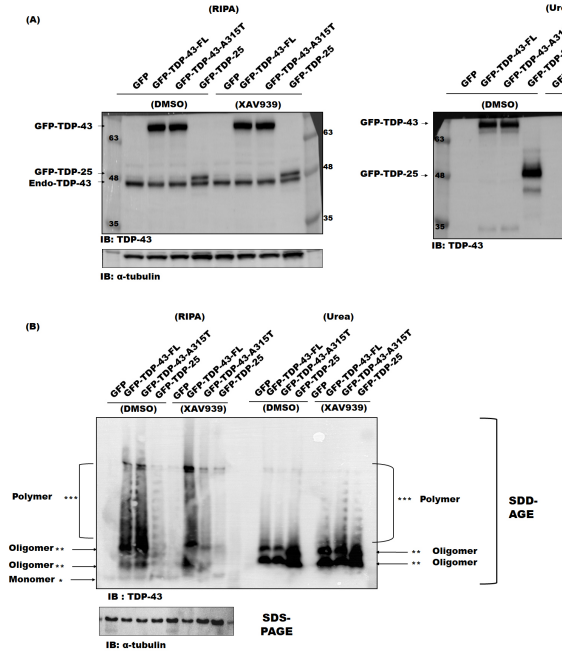

Fig. S5. (A) Relative insolubility and aggregation patterns of GFP–TDP-43-FL, the A315T mutant, and TDP-25 were assessed in the presence or absence of the TNKS-specific inhibitor XAV939. 293T cells transfected with the indicated plasmids were treated with DMSO or 10 μM XAV939 for 15 hours and then fractionated into RIPA-soluble and urea-insoluble fractions. **(B)** SDD-AGE and immunoblotting were used to detect the oligomerization and aggregates patterns of GFP–TDP-43-FL, the A315T mutant, and TDP-25. All blots were probed with TDP-43 antibody (*monomers; **oligomers, ***polymers).

Fig. S6.

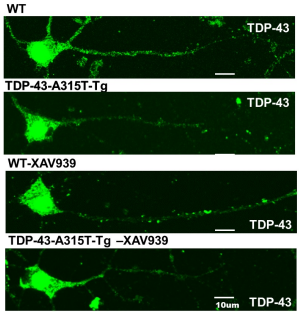

Fig. S6. TDP-43 distribution in primary motor neurons extracted from E12.5 embryos were characterized by immunofluorescence staining with TDP-43 antibody (green).
